# Gluten-Free Rice Malt Extract Powder: Pilot-Scale Production, Characterization, and Food Applications

**DOI:** 10.3390/molecules30214279

**Published:** 2025-11-03

**Authors:** Yupakanit Puangwerakul, Suvimol Soithongsuk, Kanda Wongwailikhit

**Affiliations:** 1 Faculty of Food Technology, College of Agricultural Innovation and Food Technology, Rangsit University, Pathum Thani 12000, Thailand; 2 Innovative Research and Incubation of Entrepreneur Center, Rangsit University, Pathum Thani 12000, Thailand; suvimol.88@gmail.com; 3 Department of Chemistry, Faculty of Science, Rangsit University, Pathum Thani 12000, Thailand; kanda.w@rsu.ac.th

**Keywords:** rice malt extract, pilot-scale production, microbiological media, extracted powder, enzymatic hydrolysis

## Abstract

**Background/Objectives**: This study reports pilot-scale production of gluten-free rice malt extract powder from Thai Chainat 1 rice as a sustainable alternative to barley malt extract. **Methods**: The process combined controlled malting with sequential enzymatic hydrolysis, optimized through bench-scale validation and scaled up to a 1500 L pilot system. **Results**: The resulting powder was rich in fermentable sugars (maltose 43.9 g/100 g, glucose 14.3 g/100 g), protein (5.2 g/100 g), γ-aminobutyric acid (GABA, 245.2 mg/100 g), and thiamine (0.64 mg/100 g), while free of detectable gluten, aflatoxins, and heavy metals. Microbiological quality met international safety standards. Shelf-life studies under ambient and accelerated conditions demonstrated chemical stability and bioactive retention for up to three years in laminated and HDPE packaging. Application trials confirmed that the rice malt extract powder supported yeast, bacterial, and mold growth comparably to commercial malt extract in culture media, with optimized yeast–mold agar formulations enabling direct substitution without supplementary glucose. The powder was further applied to a gluten-free malt beverage, yielding a beer-like product with acceptable physicochemical and nutritional quality, though residual alcohol levels exceeded the non-alcoholic threshold and required process optimization. **Conclusions**: Rice malt extract powder represents a safe, functional ingredient suitable for food, beverage, and industrial microbiology applications, offering opportunities to reduce import dependency and advance gluten-free innovation in emerging markets.

## 1. Introduction

As the global demand for malt extract as a food ingredient continues to rise, the intersection of supply chain resilience, dietary health, and local economic development has become increasingly critical for emerging markets. Malt extract serves as a crucial ingredient across multiple sectors, including food, beverage, and microbiological applications, playing a prominent role in baked goods, dietary supplements, animal feed, and especially beverages, both alcoholic and non-alcoholic [1]. The global malt extract market demonstrates robust growth potential, valued at USD 917.65 million in 2021 and projected to reach USD 1255.87 million by 2029 (CAGR 4.0%). The Asia–Pacific region exhibits even stronger expansion, from USD 166.42 million in 2018 to USD 272.26 million by 2027 (CAGR 5.7%), driven primarily by escalating beverage applications [2].

Thailand’s position within this expanding market presents both opportunities and challenges. While widespread consumption of malt-based beverages such as Milo and Ovaltine demonstrates significant domestic demand, Thailand’s reliance on imported barley-based malt extract creates supply vulnerabilities and elevated costs, particularly affecting small- and medium-sized enterprises that lack economies of scale [3]. This import dependency stems from climatic and agricultural constraints limiting local barley cultivation, necessitating exploration of locally abundant alternatives.

The growing global demand for gluten-free products adds critical market complexity. Gluten-related health conditions affect an estimated 1–6% of the global population, necessitating strict exclusion of gluten-containing cereals like barley [4]. The Southeast Asian gluten-free food market is experiencing rapid growth (CAGR 8.2% through 2028), driven by increasing health consciousness and diagnostic improvements [5]. This trend creates substantial opportunities for gluten-free malt extract alternatives serving both health-conscious consumers and specialized industrial applications [6].

Previous studies have explored the potential of rice and other cereals as alternatives to barley in malt extract production, mainly for brewing and gluten-free applications. For example, rice malt has been studied for its brewing suitability, but limitations such as low free amino acid content and weak enzyme activity have been noted, prompting efforts to optimize malting conditions and adjunct use [7]. Other cereal-based applications, such as rice-derived fermented foods, show promising nutritional and sensory qualities but lack comprehensive evaluations of their functional bioactives and stability compared to conventional barley malt [8].

Thai rice emerges as an ideal solution addressing these converging challenges. As one of the world’s leading rice producers, Thailand cultivates diverse varieties with functional properties suitable for industrial processing [9]. The Chainat 1 variety demonstrates favorable characteristics including high starch content (78–82%), moderate protein levels, and excellent enzymatic susceptibility, properties crucial for effective malt extract production through hydrolysis processes [10]. Unlike barley, rice is naturally gluten-free and locally abundant, offering supply security and access to expanding gluten-free markets. This approach aligns with Thailand’s agricultural diversification strategy while supporting SMEs through value-added processing that drives local innovation, strengthens rural economies, and creates new revenue streams [3].

This study addresses these gaps by developing a pilot-scale production process for gluten-free rice malt extract powder from Chainat 1 rice through optimized enzymatic hydrolysis and nutritional value. The objectives included characterizing the physicochemical and microbiological safety of the extract, evaluating its shelf life under different storage conditions, and assessing its functional performance as a substitute for commercial malt extract in microbial culture media and gluten-free beverage formulations which addresses critical industry needs by providing a scalable alternative to reduce supply chain dependencies for Southeast Asian manufacturers. Economic benefits include import substitution, agricultural diversification, and premium-priced value-added products for specialized markets.

## 2. Materials and Methods

This study conducted an experiment with pilot-scale production of malted rice using the Chainat 1 rice variety, chosen for its widespread cultivation and regional adaptability.

### 2.1. Raw Materials and Chemicals

Chainat 1 rice variety, a certified Thai cultivar (released 9 September 1993), was used as the primary raw material. This variety is characterized by its 113 cm plant height, non-photoperiod sensitivity, erect plant type, and 121–130-day maturity period with 8-week dormancy period and 12% moisture content [11]. The variety demonstrates good nitrogen fertilizer response and resistance to blast disease, brown planthopper, and white backed planthopper [12]. Rice samples were harvested in January 2024 from Pathum Thani province, Thailand, stored for 2 months post-harvest to complete dormancy. Initial paddy moisture content was around 12%, optimal for malting applications [11,12].

All chemical reagents utilized in this research were of analytical reagent (AR) grade. Acetic acid and potassium iodide were supplied by Merck Chemicals (Darmstadt, Germany). Materials such as potato starch, gamma-aminobutyric acid (GABA), iodine (used in GABA analysis), and amylose were sourced from Sigma-Aldrich Pte. Ltd., Singapore. Reagents including iodine, sodium hydroxide, sulfuric acid, sodium thiosulfate, sodium hypochlorite, petroleum ether, sodium chloride, ammonium chloride, and potassium sulfate were acquired from Ajax Finechem (Seven Hills, NSW, Australia). Fulltime (Qingdao, China) provided absolute ethanol, while 95% ethanol was obtained from Italmar (Bangkok, Thailand). Additional chemicals such as copper sulfate, glucose, lead acetate, potassium oxalate, methylene blue, boric acid, methyl red, bromocresol green, and phenolphthalein were procured from Difco Laboratory (Detroit, MI, USA). Thiamine (vitamin B1) of HPLC grade was also purchased from Sigma-Aldrich Pte. Ltd., Singapore. All chemicals were used as received, without any further purification.

Commercial enzymes were also utilized, including α-amylase (3000 U/g), β-amylase (2500 U/g), and protease (50,000 U/g). A 0.1% (*w*/*w*) addition of these enzymes corresponded to an approximate dosage of 300 U/g substrate for α-amylase, 250 U/g for β-amylase, and 5000 U/g for protease.

### 2.2. Malting Process

The initial stage involved malting, which was carried out according to the European Brewery Convention guidelines (EBC, 1987) [13]. The process began with cleaning the paddy rice and washing it in water at 30 °C for 5 min to eliminate impurities and floating kernels. This was followed by steeping, where the rice was immersed in distilled water at 30 °C for 60–72 h. The steeping water was replaced every 12 h to reduce microbial growth and ensure adequate moisture uptake by the grains. After steeping, the rice was evenly distributed in a single layer on germination trays and incubated under dark conditions at 30 °C with high relative humidity (95–100%) for 120 h. During this stage, the acrospire was allowed to develop until it reached approximately two-thirds to three-fourths of the grain length [14]. Samples (5 g) were taken every 12 h for analysis of moisture content, water activity, germination energy (GE), diastatic power (DP), and γ-aminobutyric acid (GABA). Finally, germinated rice was kiln-dried at 50–60 °C for 18–24 h to terminate enzymatic activity, yielding dry malted rice that served as the raw material for rice malt extract powder production.

### 2.3. Optimization of Extraction Process at Bench Scale

#### 2.3.1. Enzymatic Extraction Process Optimization

The optimization of enzymatic extraction was conducted through a systematic series of controlled experiments at bench scale to establish optimal conditions for maximizing soluble protein release and fermentable sugar production. The experimental design employed both exogenous enzyme supplements to facilitate comprehensive hydrolysis of starch and proteins during proteolysis and saccharification phases. A comparative experimental approach was implemented using both malted and unmalted rice samples. The inclusion of unmalted rice served as a control treatment, enabling quantitative assessment of malting-induced effects on enzymatic activity, starch hydrolysis efficiency, protein degradation rates, and bioactive compound liberation.

(1) Protease and β-Amylase Rest Phase

Malted and unmalted rice flour samples were separately prepared by mixing with distilled water at a 1:4 (*w*/*v*) ratio. Following mixture preparation, samples were heated to predetermined temperatures and supplemented with 0.1% (*w*/*w*) protease and 0.1% (*w*/*w*) β-amylase. The pH was monitored and maintained at optimal levels for the enzymes (pH 5.5–6.0) using food-grade buffer solutions when necessary. Gentle mixing was continuously applied during incubation to ensure uniform enzyme-substrate contact and heat distribution. Enzymatic extraction was subsequently performed under two temperature regimes (50 °C and 65 °C) with exposure durations of 30 and 60 min to evaluate the temperature-time interaction effects on extraction efficiency.

(2) α-Amylase Rest Phase

The slurry obtained from the protease and β-amylase treatment phase was subjected to further hydrolysis using 0.1% (*w*/*w*) commercial α-amylase [15]. Saccharification treatments were conducted at two temperature levels (55 °C and 75 °C) with incubation periods of 30 and 60 min. Optimal parameters for α-amylase-mediated saccharification were determined based on the conditions yielding maximum sugar concentration in the final extract.

The selected temperature-time combinations were based on optimal activity ranges of commercial enzymes used in extraction and saccharification (protease and β-amylase: 50–65 °C; α-amylase: 55–75 °C). Lower and higher levels were chosen to observe thermal sensitivity and efficiency shifts. These ranges also reflect typical processing conditions in industrial cereal malt and extract production, allowing evaluation of both mild and accelerated enzyme reactions.

#### 2.3.2. Validation Experiments Under Optimal Conditions

Validation experiments were conducted to demonstrate the efficacy of exogenous enzyme supplementation compared to endogenous enzyme activity alone. These experiments employed optimal conditions determined from the preceding optimization studies. A comparative analysis was performed between treatments with and without supplementary enzymes in addition to quantifying the contribution of exogenous enzymes to the overall extraction efficiency and to validate the optimized process parameters.

### 2.4. Pilot Plant Production of Rice Malt Extract Powder

Based on the optimized enzymatic extraction conditions established in Section 2.3, a scaled-up protocol was implemented at the pilot plant (1500 Liter capacity, stainless steel system). Malted Chainat 1 rice was milled into fine flour and mixed with distilled water at a solid-to-liquid ratio within the optimized ranges determined in bench-scale trials.

#### 2.4.1. Enzymatic Extraction Process

The extraction was carried out in three sequential, temperature-controlled stages designed to activate specific endogenous enzymes. In the first stage, proteolysis was induced to promote protein hydrolysis by endogenous proteases, thereby increasing the soluble protein content. The second stage involved β-amylase activity, which facilitated saccharification and the production of maltose. Finally, the third stage activated α-amylase to enhance starch hydrolysis and maximize the yield of total reducing sugars. Each stage was conducted under specific time and temperature conditions determined from the optimization study.

#### 2.4.2. Filtration, Drying, and Byproduct Utilization

Following enzymatic treatment, the slurry was filtered through muslin cloth to obtain a clarified malt extract solution. This extract was concentrated and dried using a rotary spray dryer, where the feed solution was atomized into a heated chamber via a high-speed rotating disc or nozzle. Drying air temperature and feed rate were carefully controlled to prevent heat-induced degradation of bioactive components while ensuring efficient moisture removal.

The resulting rice malt extract powder was collected, sieved for particle uniformity, and packaged in laminated pouches or high-density polyethylene (HDPE) containers for storage. Simultaneously, the solid residue was dried using hot air at 80 °C, producing a high-fiber rice flour byproduct.

### 2.5. Shelf Life Study of Rice Malt Extract Powder in Laminated Pouches and HDPE Containers

The shelf-life evaluation of rice malt extract powder was conducted using two types of packaging: clear laminated pouches and high-density polyethylene (HDPE) containers sealed with a three-layer moisture-resistant foil (aluminum/polyethylene/LDPE). Samples were stored at 30 °C (ambient), 45 °C, and 55 °C (accelerated conditions). The accelerated temperatures of 45 °C and 55 °C were selected based on industry standards for shelf-life prediction studies and Q_10_ kinetic modeling [16]. These temperatures represent realistic worst-case storage conditions in tropical climates and warehouse environments without causing unrealistic degradation mechanisms. Storage durations extended up to five months, with sampling every nine days. Shelf-life prediction was further modeled using the Q_10_ temperature coefficient based on changes in color (L* values). The parameters monitored included physical properties (color lightness, L* value), chemical properties (moisture content), antioxidant activity (DPPH radical scavenging) [17], total phenolic content [18], and vitamin B1 [19].

### 2.6. Investigation of the Application of Malt Extract Powder from Chainat 1 Variety

#### 2.6.1. Potential to Use the Malt Extract Powder from Chainat 1 Variety in Yeast and Mold Agar (YM Agar)

This study evaluated the potential of rice malt extract powder from the Chainat 1 variety as a substitute for conventional malt extract in microbiological media. The experiment followed the specifications of the benchmark commercial product, “Malt Extract for Microbiology” (Merck Chemicals). The rice malt extract powder produced in this study was incorporated into YM agar media at the same concentration as commercial malt extract for comparative testing.

The standard formulation of YM agar used in this study consisted of malt extract 3.0 g, yeast extract 3.0 g, peptone 5.0 g, dextrose 10.0 g, and agar 15.0 g per liter of distilled water [19]. Eight fungal strains, *Candida albicans* ATCC 10231, *c cerevisiae* ATCC 9763, *Saccharomyces cerevisiae* ATCC 9080, *Geotrichum candidum* DSM 1240, *Rhodotorula mucilaginosa* DSM 70403, *Penicillium commune* ATCC 10428, *Aspergillus brasiliensis* ATCC 16404, and *Trichophyton ajelloi* ATCC 28454, were used as test organisms. Inocula were prepared at initial concentrations of 10^2^–10^3^ CFU and incubated at 28 °C under standard aerobic conditions for seven days. Growth performance in the experimental YM agar (containing Chainat 1 rice malt extract powder) was compared to that in the control medium formulated with commercial malt extract. Effectiveness was determined by assessing colony formation, density, and morphology relative to the control.

#### 2.6.2. Optimization of YM Agar Formulation Using Rice Malt Extract Powder from Chainat 1

This study investigated the optimization of YM agar formulations by substituting conventional malt extract with rice malt extract powder from the Chainat 1 variety. Ten formulations (F1–F10) were prepared, with F10 (commercial YM agar containing Merck malt extract) serving as the control. The composition of each formulation per liter of distilled water is presented in Table 1. All formulations were adjusted to a final pH of 6.2 ± 0.2 at 25 °C and sterilized by autoclaving at 121 °C for 15 min.

The microbial strains used in this study included *Aspergillus niger* TISTR5161 (filamentous fungus), *Saccharomyces cerevisiae* TISTR5161 (yeast), *Bacillus subtilis* TISTR001 (Gram-positive bacterium), and *Escherichia coli* TISTR780 (Gram-negative bacterium). Fresh cultures were prepared from standard media and adjusted to inoculum concentrations of 10^2^–10^3^ CFU/mL prior to use. Each strain was inoculated in triplicate onto YM agar plates by spreading 100 µL of inoculum onto the surface of the medium. Plates were incubated at 28 °C for fungi and yeast, and at 35 °C for bacteria. Growth was evaluated qualitatively by observing colony morphology and density, and quantitatively by measuring colony diameter (for fungi and yeast) or enumerating CFUs (for bacteria) at designated time intervals (24, 48, and 72 h for bacteria and yeast; longer periods as appropriate for fungi).

Effectiveness of the experimental media was defined as showing no statistically significant differences in colony growth (size, number, or morphology) compared with the control formulation (F10).

#### 2.6.3. Preparation of Wort and Non-Alcoholic Beer Using Rice Malt Extract Powder from the Chainat 1 Variety

Low-sugar wort was prepared following the method of Puangwerakul et al. (2014) [20] with minor modifications. Malt extract powder was mixed with water at a ratio of 1:11 (*w*/*v*), yielding a wort with an initial sugar content of 8°Plato. Fermentation was initiated by inoculating *Saccharomyces cerevisiae* RSU No. 10 yeast and incubating at 30 °C for 14 days. To reduce the alcohol content to below 0.5% (*v*/*v*), the fermented beer was subjected to vacuum evaporation. This process was conducted at 80 °C for 2 h. The resulting dealcoholized beer was then filled into containers and pasteurized prior to subsequent analysis. The yeast strain *Saccharomyces cerevisiae* RSU No. 10, obtained from the Culture Collection of Rangsit University, was selected for fermentation due to its moderate ethanol production and favorable metabolic profile.

### 2.7. Analytical Methods for Nutritional, Functional, and Safety Evaluation

All measurements were performed in triplicate.

#### 2.7.1. Germination Energy

Germination energy was assessed following the standard procedure outlined by the European Brewery Convention (EBC, 1987) [13]. One hundred intact, unbroken paddy rice grains were arranged in 90 mm Petri dishes lined with two layers of Whatman No.1 filter paper, moistened precisely with 8 mL of distilled water. The samples were incubated in complete darkness at 20 °C for a period of three days. GE (%) was calculated as:

GE (%) = (Number of germinated seeds)/(Total seeds) × 100 (1)

#### 2.7.2. Diastatic Power Analysis

Diastatic power was determined according to the European Brewery Convention method (EBC, 1987) [13]. A 21 g sample of ground malted rice was combined with 500 mL of distilled water and incubated in a mashing bath at 40 °C for 60 min, with continuous stirring to enhance enzymatic starch breakdown. After the incubation, the mash was cooled to ambient temperature and filtered through Whatman No. 1 paper to obtain a clear filtrate for analysis. The assay employed an acetate buffer (pH 5.0), a 1% soluble starch solution (in 500 mL distilled water), and an iodine reagent prepared by dissolving 12.7 g of iodine and 20 g of potassium iodide in 1 L of water. Additional analytical-grade reagents included sodium hydroxide, sulfuric acid, and sodium thiosulfate. The analytical procedure involved combining the enzyme extract with the starch solution under standardized conditions, followed by an iodine assay to assess starch degradation. Reducing sugars formed during the reaction were subsequently quantified through titration. The diastatic power was calculated based on the volume differences between blank (V_B_) and test (V_T_) titrations using the following:

DP_1_ = F(V_B_ − V_T_) (2)

DP_2_ = (DP_1_/(100 − M)) × 100 (3)

where: V_B_, V_T_ = volumes for blank and test; F = correction factor; M = moisture content (%).

#### 2.7.3. Free Amino Nitrogen (FAN)

Free Amino Nitrogen (FAN) was quantified through a colorimetric assay utilizing Ninhydrin-based reagents according to the European Brewery Convention method (EBC, 1987) [13]. The procedure involved diluting the sample, combining it with a prepared color reagent, and incubating the mixture at 20 °C for a fixed period. After incubation, the absorbance was measured at 520 nm using a spectrophotometer. FAN concentration (mg/L) was determined based on the absorbance values using a standard calibration curve.

FAN (mg/L) = (A_s_ − A_b_ − A_d_)/A_g_ × 2 × d (4)

where A_s_ = sample absorbance, A_b_ = Blank, A_d_ = dark sample correction, A_g_ = absorbance of 2 mg/L glycine standard, d = 2 mg/L.

#### 2.7.4. DPPH Radical Scavenging Assay

The assay was performed following Brand-Williams et al. (1995) [17] with minor modifications [21]. Extracts (100 µL; 0.005–5 mg/mL) were mixed with 100 µL of DPPH solution (0.8 mg/mL; Sigma-Aldrich, St. Louis, MO, USA) and incubated in the dark for 30 min. Absorbance was measured at 517 nm, and scavenging activity was calculated as:

%DPPH radical scavenging = (A_blank_ − A_extract_)/A_blank_ × 100 (5)

where A_blank_ is the absorbance without extract and A_extract_ is the absorbance with extract.

#### 2.7.5. Iodine Blue Value

Amylose content was determined using the iodine blue value method [22]. A standard iodine reagent was prepared by dissolving iodine in 95% ethanol and diluting it with 1 M sodium hydroxide (NaOH). The rice sample reacted with this iodine solution, and the resulting blue complex was measured spectrophotometrically at 620 nm. A calibration curve was established using amylose standards ranging from 8% to 40% to quantify amylose concentration in the samples.

#### 2.7.6. Moisture Content Analysis

Moisture content in rice grains was measured using the standard oven-drying technique according to Association of Official Analytical Chemists (AOAC) method 925.10 (AOAC, 2000) [23]. About 5 g of homogenized rice sample were placed in a pre-weighed, pre-dried aluminum moisture dish and dried in a hot air oven at 105 ± 2 °C for 24 h. After drying, the samples were allowed to cool in a desiccator for 30 min before being reweighed. Moisture percentage was then calculated based on the difference in weight before and after drying.

Moisture Content (%) = (W_1_ − W_2_)/W_1_ × 100 (6)

where W_1_ is the initial weight of the sample (g) and W_2_ is the final weight after drying (g).

#### 2.7.7. Ash Content

Ash content was determined according to AOAC 920.153 (AOAC, 2000) [23]. A sample weighing 5 g was placed in a clean crucible and incinerated in a muffle furnace at 500–550 °C until all organic matter was fully combusted, leaving behind a consistent white ash. The crucible was then cooled in a desiccator to room temperature before being reweighed. Ash content was calculated based on the residual weight of mineral matter. Ash content was calculated: %Ash = (Ash weight/Sample weight) × 100.

Ash (%) = W_a_/W_s_ × 100 (7)

where W_a_ (g) is the weight of ash and W_s_ (g) is weight of sample.

#### 2.7.8. Crude Protein Content

Crude protein content was measured using the Kjeldahl method, in accordance with AOAC Official Method 984.13 [23]. Approximately 5 g of homogenized sample were digested with 15 mL of concentrated sulfuric acid (98% H_2_SO_4_) and 10 g of a catalyst mixture (potassium sulfate, copper sulfate, and selenium in a 100:10:1 *w*/*w* ratio) at 420 °C for 90 min. After digestion, the mixture was cooled and diluted to a final volume of 100 mL with deionized water. The diluted digest was then distilled with 40% sodium hydroxide (NaOH), and the released ammonia was captured in a 2% boric acid (H_3_BO_3_) solution. The amount of ammonia was quantified by titration with 0.05 M sulfuric acid (H_2_SO_4_), using a mixed indicator composed of methyl red and bromocresol green (1:5 *v*/*v*). Crude protein content was calculated as:

Crude protein (%) = ((V_s_ − V_b_) × M × 14.01 × 6.25)/W_s_ × 100 (8)

where V_s_ (mL) is the titrant volume for sample, V_b_ (mL) is the titrant volume for blank, M is the molarity of H_2_SO_4_, 14.01 is the atomic weight of nitrogen, and 6.25 is the nitrogen-to-protein conversion factor for cereals. W_s_ is the weight of sample.

#### 2.7.9. Crude Fat Content

Crude fat content was determined using the Soxhlet extraction technique [23,24]. Between 3 and 4 g of dried sample were placed into pre-weighed, fat-free extraction thimbles and extracted continuously with petroleum ether for 16 h. After extraction, the solvent was evaporated, and the receiving flask was dried in an oven at 100 °C until a constant weight was obtained. The crude fat content was calculated gravimetrically from the difference in flask weights before and after extraction.

Fat (%) = (W_f_/W_s_) × 100 (9)

where W_f_ (g) is weight of fat and W_s_ (g) is weight of sample.

#### 2.7.10. Crude Fiber Content

Crude fiber content was determined by sequential acid and alkaline digestion. Three grams of the defatted sample were first boiled with 0.255 N sulfuric acid (H_2_SO_4_), followed by treatment with 0.313 N sodium hydroxide (NaOH). The resulting residue was filtered, thoroughly washed with distilled water, dried, and then incinerated in a muffle furnace. Crude Fiber content was calculated as the weight difference between the dried residue and the ash, representing the organic fibrous material lost during ashing according to AOAC procedures [23].

#### 2.7.11. Available Carbohydrate

The percentage of available carbohydrate was determined by the difference method according to AOAC (2000) [23]. This value was calculated by subtracting the percentages of moisture, protein, fat, ash, and crude fiber from 100%, as shown in the following formula:

Available Carbohydrate (%) = 100 − (%Moisture + %Protein + %Fat + %Ash + %Fiber)(10)

#### 2.7.12. Total Energy

The caloric value was calculated using Atwater conversion factors [23], where protein and carbohydrate were assigned 4 kcal/g and fat 9 kcal/g. The total energy content (kcal/100 g sample) was estimated as:

Energy (kcal/100 g) = (4 × Protein) + (9 × Fat) + (4 × Carbohydrate)(11)

#### 2.7.13. Total and Reducing Sugar Analysis

Sugar content was analyzed using the classical Fehling’s titration technique. To clarify the sample extracts, 10% neutral lead acetate and 10% potassium oxalate were added. The clarified solution was then divided for separate analysis of reducing and total sugars. Titration was performed using Fehling’s A and B solutions, with 1% methylene blue employed as the endpoint indicator as specified in AOAC (2000) [23].

#### 2.7.14. Free Fatty Acid (FFA)

FFA were determined by homogenizing 20 g of the sample with 50 mL of hot water, followed by filtration to obtain a clear extract. A 20 mL aliquot of the filtrate was then mixed with a few drops of phenolphthalein indicator and 95% ethanol. The mixture was titrated with 0.1 N sodium hydroxide (NaOH) until a persistent pink endpoint was observed. The FFA content was calculated based on the volume of NaOH used in the titration according to AOAC (2000) [23] using the following equation:

FFA (%) = ((V_s_ − V_b_) × N × 28.2 × 2.5)/W_s_ × 100 (12)

where V_s_ (mL) is the titrant volume for sample, V_b_ (mL) is the titrant volume for blank, N is the normality of NaOH, W_s_ is the weight of sample.

#### 2.7.15. Total Phenolic Content (TPC)

Determined by the Folin–Ciocalteu colorimetric method using gallic acid as a standard [18]. Results were expressed as mg gallic acid equivalents (GAE) per g of sample.

#### 2.7.16. Soluble Fiber Content

Soluble fiber content was determined using the enzymatic-gravimetric method according to AOAC 991.43 [23], involving sequential enzymatic treatment with α-amylase, protease, and amyloglucosidase, followed by ethanol precipitation and gravimetric measurement.

#### 2.7.17. Determination of GABA

The gamma-aminobutyric acid (GABA) content was analyzed following the method described by Loikaeo et al. (2024) [25] and Wijngaard et al. (2005) [26]. A 5-g sample was mixed with 5 mL of 80% ethanol and incubated for 2 h to allow for extraction. The mixture was then filtered, and a subsequent extraction was performed using 3 mL of water and Whatman No. 4 filter paper. Sequentially, borate buffer, 1000 µL of phenol reagent, and 400 µL of sodium hypochlorite were added to the extract. The reaction mixture was then heated at 100 °C for 10 min. After cooling, ethanol was added, and the absorbance was measured at 630 nm to quantify GABA content.

#### 2.7.18. Color Measurement

Color evaluation was performed with a chromameter (Minolta CR-10) to record the L* (lightness), a* (red–green), and b* (yellow–blue) parameters of the samples [27].

#### 2.7.19. Determination of Vitamin B1

Thiamine (vitamin B1) content was analyzed using high-performance liquid chromatography (HPLC) with a SHIMADZU LC10AD system (Shimadzu Corporation, Kyoto, Japan) according to Liu (2002) [19]. Approximately 0.2–0.5 g of the sample was hydrolyzed in 0.1 N hydrochloric acid at 121 °C for 30 min. The hydrolysate was then adjusted to a pH range of 4.0–4.6 using a sodium acetate buffer. Enzymatic digestion was performed by adding taka diastase at a concentration of 0.1 mL per gram of sample, followed by incubation at 40 °C for 3 h. The resulting solution was filtered and injected into the HPLC system for quantification.

Chromatographic separation was achieved on a Mightysil RP-18 GP Aqua column (250 mm × 4.6 mm i.d., 5 µm; Kanto Chemical Co., Tokyo, Japan) at 35 °C with a flow rate of 0.5 mL/min. The gradient program was from 0 to 30 min, 0.3 to 100% B. Detection was performed at 220 nm using a UV detector.

#### 2.7.20. Determination of Vitamins B2, B3, B5, B6, and B9

The concentrations of vitamins B2 (riboflavin), B3 (niacin), B5 (pantothenic acid), B6 (pyridoxine), and B9 (folic acid) were determined by Central Laboratory (Thailand) Co. (Bangkok, Thailand), Ltd., an ISO/IEC 17025-accredited laboratory [28]. The results are presented based on single replicate analyses, as documented in the Certificates of Analysis under Report Nos. TRBK68/22150.

#### 2.7.21. Mineral Analysis

Minerals (Na, K, Ca, Mg) and Heavy Metals (Pb, Cd, As, Hg, Sn) were determined by Central Laboratory (Thailand) Co., Ltd., an ISO/IEC 17025-accredited laboratory. The results are presented based on single replicate analyses. Determination was done by inductively coupled plasma–mass spectrometry (ICP–MS) following AOAC 985.16 [23].

#### 2.7.22. Microbiological Analysis

Microbiological Quality: Total plate count, yeast and mold count, and coliforms were determined using standard plate count methods [29]. Results are expressed as CFU/g.

#### 2.7.23. Contaminant Analysis

Contaminant analysis was performed to evaluate the presence of mycotoxins (aflatoxins), gluten allergen, and heavy metals. All analyses were conducted by Central Laboratory (Thailand) Co., Ltd., an ISO/IEC 17025-accredited facility. The results were obtained from single replicate measurements and are reported in the Certificates of Analysis (Report No. TRBK68/00027 Part 1).

#### 2.7.24. Amino Acid Composition

Amino acid composition was analyzed by Central Laboratory (Thailand) Co., Ltd., an ISO/IEC 17025-accredited laboratory. The results were based on single replicate measurements and reported under Certificate of Analysis No. TRBK68/00028. Quantification was performed using validated in-house protocols.

#### 2.7.25. Xylose Determination

Xylose content was determined by high-performance liquid chromatography with refractive index detection (HPLC-RI). The sample (1 g) was dissolved in 10 mL of deionized water, filtered through a 0.45 μm syringe filter, and analyzed using an Agilent Hi-Plex Ca column (Agilent Technologies, Bangkok, Thailand) at 85 °C [30].

#### 2.7.26. Phytic Acid Determination

Phytic acid content was determined using the colorimetric method of Haug and Lantzsch (1983) [31], based on ferric ion–bipyridine complex formation. One gram of sample was extracted with 0.2 N HCl (100 mL) for 10 min, followed by reaction with ferric solution, heating in boiling water for 30 min, cooling, and centrifugation at 6500 rpm for 30 min. The supernatant was mixed with 2,2′-bipyridine solution, and absorbance was measured at 519 nm. Phytic acid concentration was calculated from a standard calibration curve [31].

### 2.8. Statistical Analysis

All experiments were conducted in triplicate. Statistical analysis was performed using SPSS version 29. Analysis of variance (ANOVA) was used to assess differences among means, and significant differences were determined using Tukey’s post hoc test at *p* < 0.05.

## 3. Results and Discussion

### 3.1. Characteristics of Malted Chainat 1 Rice Variety

**Figure 1:**
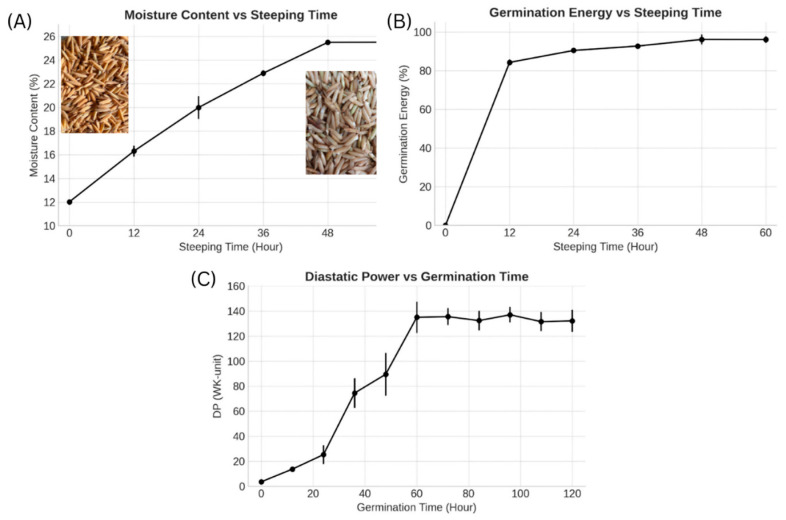
Changes in (**A**) moisture content (%), (**B**) germination energy (%), and (**C**) diastatic power (WK units) of Chainat 1 rice during the 120-h steeping and germination period (n = 3).

The Chainat 1 rice variety underwent germination for 5 days (120 h), during which samples were collected every 12 h to monitor malting progress. Optimal steeping and germination durations were observed at 48 and 60 h, respectively. While steeping, moisture content rose rapidly, peaking at 25.50 ± 0.25% by 48 h, indicating the grains had reached moisture equilibrium necessary to trigger germination (Figure 1A). This aligns with the findings of Kamjijam et al. (2021) [32] who reported similar moisture dynamics during malting. Germination energy (GE), a measure of the seed’s ability to germinate under controlled conditions, reached 97 ± 1.0%, satisfying the European Brewery Convention (EBC, 1987) standard [13], which requires a minimum of 95% (Figure 1B). A high GE indicates that rice grains possess strong viability and uniform germination potential, crucial for consistent enzyme development during malting [33]. In practical terms, this ensures effective transformation of starches and proteins into fermentable and soluble forms, enhancing malt quality and extract yield. Diastatic Power (DP), which reflects the malt’s enzymatic activity, especially its ability to break down starch into fermentable sugars, peaked at 135 ± 12.5 WK-units at 60 h (Figure 1C). This value falls within the acceptable range for malted rice (106–194 WK-units), confirming strong enzymatic potential for saccharification.

Comparative nutritional analysis of unmalted and malted Chainat 1 rice (Table 2) revealed key compositional changes post-malting. Amylose content decreased from 29.31 ± 1.20% to 27.88 ± 0.21%, while carbohydrate content slightly declined, supporting previous findings that carbohydrates serve as the primary energy source during germination [32,33]. Protein content increased significantly from 6.95 ± 0.05 to 8.88 ± 0.00 g/100 g, and crude fiber rose from 2.00 ± 0.56 to 5.78 ± 0.55 g/100 g. The apparent increase in crude fiber content may result from the concentration effect due to the formation of resistant compounds during germination, plus the development of cell wall modifications that become less soluble in the acid-alkaline digestion procedure used in the AOAC crude fiber method [34]. A substantial increase in total sugar (from 2.02 to 11.54 g/100 g), including maltose (3.30 g/100 g), reflects starch hydrolysis during germination [32]. Lipid content remained largely unchanged, while gamma-aminobutyric acid (GABA) levels rose nearly fivefold [35], from 2.05 ± 0.17 to 10.63 ± 0.22 mg/100 g. This enhancement is consistent with other studies on sprouted grains and supports the nutritional enrichment potential of malted rice [3]. Vitamin and Mineral amounts found were used as baseline for comparison with the product of malt extract [33].

### 3.2. Bench-Scale Optimization of Enzymatic Extraction from Unmalted and Malted Chainat 1 Rice

The enzymatic extraction process of Chainat 1 rice was investigated under varying temperature and time conditions to determine the optimal conditions for maximizing enzymatic extraction yield. Both malted and unmalted rice were compared to evaluate the contribution of malting to enzymatic activity. In this study, reducing sugar content was selected as the primary parameter for determining optimal extraction conditions, as it directly reflects the efficiency of enzymatic starch hydrolysis and is critical for downstream applications in fermentation and microbiological usage. The results obtained from the two stepwise phases are as follows.

#### 3.2.1. Protease and β-Amylase Rest Phase

This extraction stage aimed to enhance maltose release through the activity of protease and β-amylase present in malted rice both from endogenous and exogenous enzyme (supplemental amount of 0.1% protease and 0.1% β-amylase). Samples were incubated at two temperatures (50 °C and 65 °C) for 30 or 60 min. The amount of reducing sugar was reported in Table 3.

Table 3 shows that malted rice released significantly higher amount of reducing sugars than unmalted rice in all treatments, confirming that malting effectively activates β-amylase. The highest reducing sugar was obtained at 50 °C in 60 min (7.30 ± 0.02 g/100 g). By contrast, higher temperature (65 °C) reduced sugar yield, indicating thermal sensitivity of β-amylase. Therefore, 50 °C for 60 min was identified as the optimal β-amylase rest condition in malted Chainat 1 rice.

#### 3.2.2. α-Amylase Rest Phase

The subsequent phase via endo- and exogenous α-amylase activity is reported in Table 4.

It was clearly observed that only malted rice produced appreciable amounts of reducing sugars, whereas the unmalted samples showed no significant change under any condition. The yield of reducing sugars from the hydrolysis of malted rice increased significantly with both higher temperature and longer incubation. The maximum reducing sugar content (14.89 ± 0.13 g/100 g) was obtained at 75 °C for 60 min, indicating that α-amylase is relatively heat-tolerant and exhibits time-dependent hydrolytic activity [36]. Therefore, the optimal α-amylase rest condition was determined to be 75 °C for 60 min.

In summary, based on results from Table 3 and Table 4, the optimized extraction protocol for producing malt extract from Chainat 1 rice consists of two sequential enzymatic phases: (1) Protease and β-amylase rest at 50 °C for 60 min, enhancing protein hydrolysis and maltose release. (2) α-amylase rest at 75 °C for 60 min, maximizing starch hydrolysis and total reducing sugar yield. This stepwise protocol demonstrates that malting significantly enhances enzymatic activity and sugar recovery compared with unmalted rice.

#### 3.2.3. Validation Batch Experiments Under Optimal Extraction Conditions

Validation experiments were conducted in a one-pot system under the optimized enzymatic extraction process, covering both the protease/β-amylase and α-amylase rest phases. Soluble protein and fermentable sugar yields were compared between treatments using endogenous enzymes alone and those supplemented with exogenous enzymes. The treatments were designated as follows: T1 served as the control with no enzyme supplementation, relying solely on endogenous activity; T2 involved supplementation with 0.1% protease and 0.1% β-amylase at 50 °C; T3 applied the same enzyme combination as T2 but excluded α-amylase treatment at 75 °C; and T4 represented full enzyme supplementation, including α-amylase treatment at 75 °C.

The results (Table 5) showed that enzyme supplementation significantly improved the extract’s nutritional composition. The addition of protease and β-amylase at 50 °C (T2) nearly doubled the soluble protein content and increased maltose levels by 2.4-fold compared to the control (T1). When the temperature subsequently increased to 75 °C, the introduction of α-amylase (T4) further enhanced sugar hydrolysis, resulting in a 2.8-fold increase in maltose, a 2.9-fold increase in glucose, and the emergence of previously undetectable sugars such as sucrose and fructose. Xylose levels also rose markedly.

These findings indicate that sequential addition of protease/β-amylase at moderate temperature and α-amylase at higher temperature significantly enhances the extraction of fermentable sugars and soluble proteins.

### 3.3. Production of Rice Malt Extract Powder

**Figure 2:**
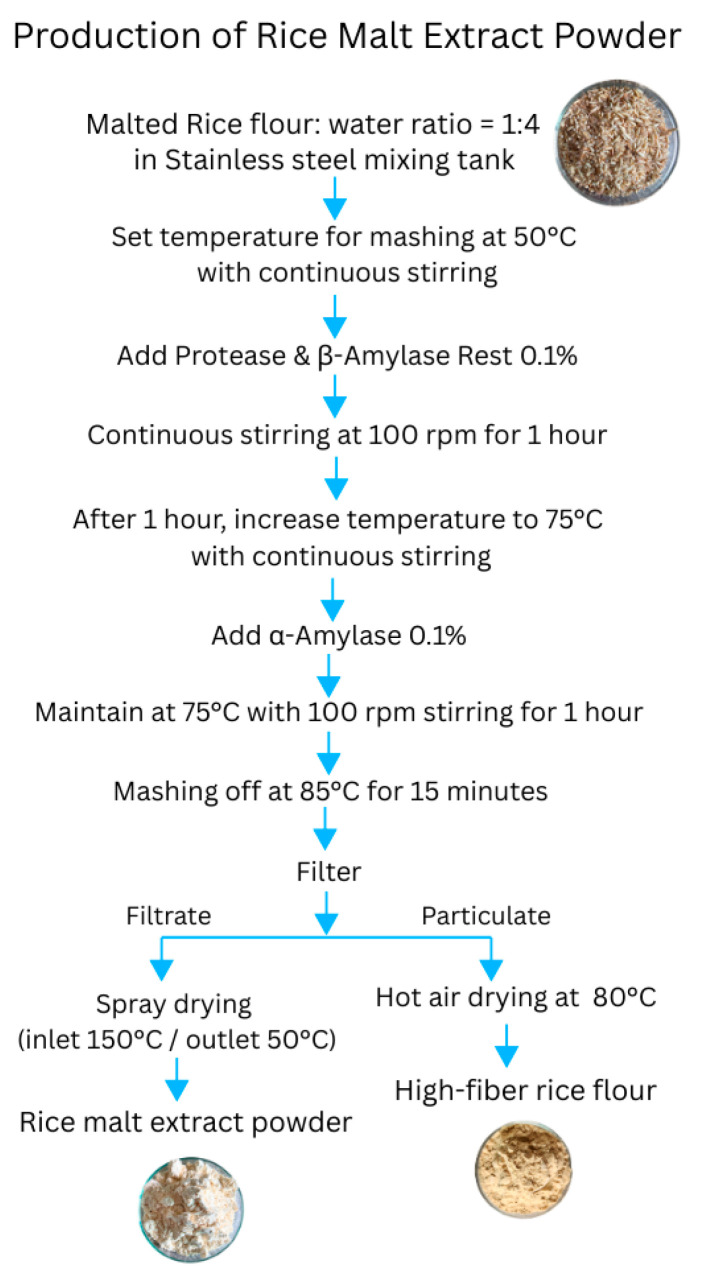
Process flow diagram for the production of rice malt extract powder from malted Chainat 1 rice.

Using the optimized enzymatic extraction conditions, production of rice malt extract powder from Chainat 1 rice was scaled up in a 1500-L pilot-scale facility. The process (Figure 2) began with milling malted rice into flour, mixing it with distilled water (1:4 *w*/*v*), and heating to 50 °C under agitation. At this stage, 0.1% (*w*/*w*) protease and β-amylase were added and incubated for 60 min, followed by heating to 75 °C with α-amylase supplementation for another 60 min. Enzyme activity was terminated by heating to 85 °C for 15 min, after which the mash was filtered to obtain a clarified liquid extract.

**Figure 3:**
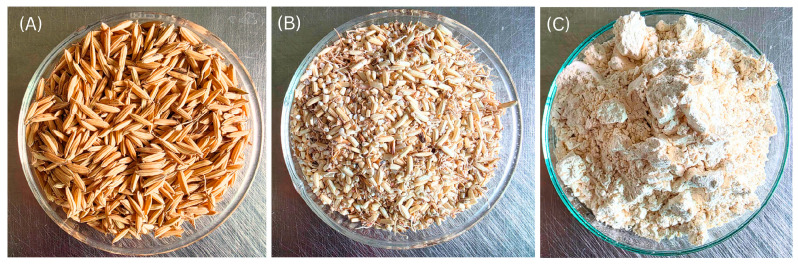
Visual representation of rice malt extract powder development: (**A**) Chainat 1 paddy rice before malting, (**B**) malted rice, and (**C**) final rice malt extract powder.

The extract was spray-dried at inlet/outlet temperatures of 150 °C/50 °C to preserve bioactive components, yielding rice malt extract powder that was sieved and packaged in laminated pouches or HDPE containers. The fibrous residue was dried at 80 °C to produce a high-fiber flour byproduct, though this was not investigated further in the present study. Visual examples of raw rice, malted grains, and the final powder are shown in Figure 3, illustrating the transformation into a soft, functional powder suitable for food and microbiological applications.

#### 3.3.1. Nutritional Analysis of the Extracted Rice Malt Powder

The rice malt extract powder derived from malted Chainat 1 rice exhibited a robust nutritional profile (Table 6). Carbohydrates dominated the composition (88.9 ± 6.4 g/100 g), with sugars accounting for over 64%, notably maltose (43.85 g/100 g) and glucose (14.31 g/100 g). This sugar composition reflects the efficiency of sequential β- and α-amylase hydrolysis steps in converting starch to fermentable disaccharides and monosaccharides (Section S1, Supplementary Materials: HPLC profiles confirm the efficient conversion of rice starches into fermentable sugars during the enzymatic extraction process). Functionally, the powder was notably rich in γ-aminobutyric acid (GABA, 245.20 ± 2.55 mg/100 g) and total phenolic compounds (25.59 ± 0.26 mg GAE/100 g). GABA is a bioactive compound linked to calming and neuroprotective effects, while phenolics contribute antioxidant activity, supporting shelf stability and potential health claims [32]. The presence of vitamin B1 (0.64 ± 0.07 mg/100 g) adds further value, as it is essential for carbohydrate metabolism and nervous system support, traits particularly relevant for energy-dense or microbiological media formulations [35]. Low fat (0.48 g/100 g) and moisture content (1.74%) contribute to the product’s long shelf life and stability.

Mineral analysis revealed that the rice malt extract powder contained adequate levels of essential electrolytes, including potassium (73.85 ± 4.15 mg/100 g) and sodium (39.46 ± 5.02 mg/100 g), which support cellular function and osmotic balance. The extract was also a moderate source of magnesium (91.96 ± 5.55 mg/100 g) and calcium (161.76 ± 15.2 mg/100 g), key cofactors in enzymatic reactions and bone health [36]. These values are comparable to or exceed typical levels found in commercial malt extracts, reinforcing their potential as a nutritionally competitive alternative [15].

The amino acid composition (Table 7) highlighted glutamic acid (863.49 mg/100 g) and leucine (358.62 mg/100 g) as predominant constituents. Glutamic acid enhances umami flavor, while leucine supports muscle protein synthesis. Other notable amino acids included arginine (388.36 mg/100 g) and alanine (264.68 mg/100 g), contributing to both nutritional and functional value. Several essential amino acids (e.g., threonine, methionine, tyrosine) were present at low or undetectable levels, consistent with known limitations of rice-based protein sources [32]. Nonetheless, the presence of multiple free amino acids supports the extract’s utility in microbiological culture media and specialized food formulations [39,40].

Overall, the nutritional analysis from Table 6 and Table 7 confirms that the rice malt extract powder derived from Chainat 1 rice is rich in fermentable sugars, essential amino acids, bioactive compounds, and micronutrients, making it a competitive alternative to conventional barley-based malt extract for both food and microbiological applications.

#### 3.3.2. Microbiological and Contaminant Analysis of Malt Extract Powder

To ensure the safety and suitability of the rice malt extract powder for food and microbiological applications, a comprehensive analysis of microbiological quality and contaminant levels was conducted.

The microbiological evaluation (Table 8) confirmed that all tested parameters, including total yeast and mold, *Staphylococcus aureus*, *Bacillus cereus*, *Clostridium perfringens*, Coliforms, *Escherichia coli*, and *Salmonella* spp., were either undetectable or within the acceptable limits specified for food-grade and analytical-grade malt extracts. These results affirm the hygienic quality and microbial safety of the product under standard production conditions [42].

Further analysis addressed the presence of potential contaminants, including mycotoxins, gluten allergens, and heavy metals (Table 9). No aflatoxins (B1, B2, G1, G2) were detected, indicating the absence of fungal toxin contamination during production and storage. Similarly, gluten allergen levels were below the detection limit, confirming the product’s gluten-free status, an important attribute for allergen-sensitive consumers and specific microbiological media formulations [36]. The undetectable levels of heavy metals reflect both controlled processing conditions and the low inherent heavy metal content of Chainat 1 rice, supporting the product’s suitability for food applications with international safety standards [48].

Together, these findings demonstrate that the malt extract powder from Chainat 1 rice meets essential safety and purity criteria for commercial applications. Its microbial integrity and absence of harmful residues further validate its use in sensitive microbiological procedures and clean-label food products [39].

### 3.4. Shelf-Life Evaluation of Rice Malt Extract Powder

The shelf life of rice malt extract powder was assessed by monitoring key physicochemical parameters under different packaging and storage conditions. Two packaging types were tested: clear laminated pouches and high-density polyethylene (HDPE) containers. Samples were stored under ambient (30 °C) and accelerated (45 °C and 55 °C) conditions and evaluated at nine-day intervals for changes in color lightness (L*), moisture content, total phenolic content, antioxidant activity (DPPH scavenging), and vitamin B1. The most sensitive parameter showing the earliest significant changes was identified for each packaging type to serve as the limiting factor for shelf-life prediction.

*Laminated Pouches:*

Among all monitored parameters (color lightness L*, moisture content, total phenolic content, and DPPH antioxidant activity), color lightness (L*) was the first parameter to show significant changes over time in laminated pouches, making it the critical limiting factor for shelf-life determination. Both moisture content and total phenolics remained stable, even under accelerated conditions. For instance, moisture content remained below 2% throughout storage at 30 °C, suggesting excellent barrier properties and shelf stability. These findings indicate that the powder’s antioxidant integrity was largely preserved despite elevated temperatures, consistent with results from Weeragul et al. (2024) [40]. Full data are presented in Table S1 (see Supplementary Materials, Section S2).

**Figure 4:**
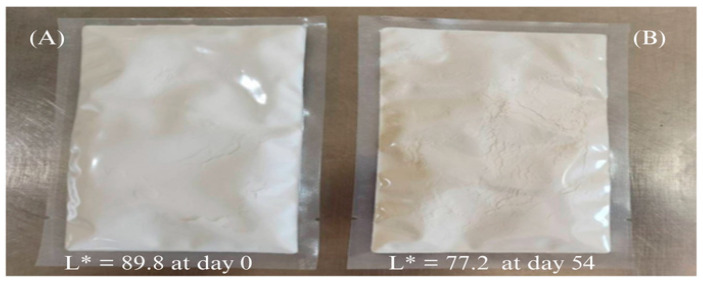
Visual changes in rice malt extract powder during accelerated storage at 55 °C: (**A**) Appearance at day 0 and (**B**) appearance after 54 days. The browning effect reflects non-enzymatic Maillard reactions, consistent with the decline in L* color values observed under high-temperature conditions.

Changes in L* values under elevated temperatures were used to model shelf life. As shown in Table 10, no significant degradation was observed at 30 °C. However, a marked decline in L* was recorded at 45 °C and 55 °C, attributed to non-enzymatic browning reactions, such as the Maillard reaction [25,26], resulting in perceptible browning (see Figure 4). Using a Q_10_-based predictive model, the estimated shelf life based on color change at 30 °C was approximately 534 days, supporting its viability for long-term storage in food or microbiological applications [49].

*HDPE Containers:*

For samples stored in HDPE containers, comprehensive analysis of the same parameters (color lightness L*, moisture content, total phenolic content, and vitamin B1) revealed that vitamin B1 was the most sensitive parameter, showing significant degradation before other quality indicators. While color changes (L*) were also monitored, vitamin B1 degradation occurred earlier and was therefore selected as the limiting factor for shelf-life prediction in this packaging system. At accelerated temperature of 55 °C, vitamin B1 levels declined from 0.64 mg/100 g to 0.40 mg/100 g over 180 days (Table 11). In contrast, levels remained stable under ambient conditions (30 °C). This trend aligns with the known thermal lability of vitamin B1, particularly under dry heat [50]. Using first-order degradation kinetics and Q_10_ modeling, the predicted shelf life for vitamin B1 retention was approximately 1095 days (3 years) at 30 °C. Full vitamin stability data are provided in Table S2, (see Supplementary Materials, Section S3).

The different critical parameters for each packaging type reflect the distinct barrier properties and protective mechanisms of the materials. Laminated pouches showed greater susceptibility to light-induced color changes due to their transparency, while HDPE containers provided better color protection but allowed greater vitamin B1 degradation, likely due to differences in oxygen permeability. Overall, both packaging systems provided effective protection for rice malt extract powder under normal storage conditions, with predicted shelf lives of up to 3 years at ambient temperature based on their respective limiting quality parameters. These results support the product’s commercial viability with appropriate packaging selection based on the intended storage environment and quality priorities.

It should be noted that both vitamin B1 degradation and Maillard browning reactions occur in both packaging systems under elevated temperatures [36]. However, the relative rates of these degradation pathways differ due to the distinct barrier properties of each material. Laminated pouches, being more transparent, showed accelerated color changes that preceded significant vitamin B1 losses. Conversely, HDPE containers provided better light protection but exhibited faster vitamin B1 degradation, likely due to differences in oxygen permeability. The limiting factor for shelf-life prediction was therefore determined by which degradation mechanism reached significant levels first in each packaging system.

### 3.5. Investigation of the Application of Malt Extract Powder

This experiment aimed to evaluate the practical applications of pilot-scale rice malt extract powder through three assessments: its use as a nutrient source for microbial cultivation, as a component of yeast and mold agar media, and as a base for a gluten-free, non-alcoholic malt beverage. These studies benchmarked its performance against commercial alternatives to demonstrate its potential in food and microbiological industries.

#### 3.5.1. Evaluation of Malt Extract Powder as a Substrate for Fungal Cultivation

This section examines the efficacy of rice malt extract powder as a substrate for the cultivation of diverse fungal microorganisms, with a direct comparison to a leading commercial malt extract powder (Merck “Malt Extract for Microbiology”). Eight strains of fungi, including *Candida albicans*, two *Saccharomyces cerevisiae* strains, *Geotrichum candidum*, *Rhodotorula mucilaginosa*, *Penicillium roquefortii*, *Aspergillus niger*, and *Trichoderma harzianum*, were inoculated onto solid agar media prepared with either the rice malt extract powder or the commercial counterpart. The initial inoculum was set at 10^2^–10^3^ CFU, and cultures were incubated at 28 °C for seven days.

Results summarized in Table 12 show that for the first five strains, colony counts on rice malt extract powder media were statistically indistinguishable from those on commercial malt extract, indicating the suitability of rice malt extract powder as a substitute in supporting the growth of yeasts and certain fungi (*p* ≥ 0.05). Conversely, for the filamentous fungi *Penicillium roquefortii*, *Aspergillus niger*, and *Trichoderma harzianum*, the rice-derived media yielded significantly lower colony counts compared to commercial media, suggesting possible limitations in nutrient composition that may not fully meet the metabolic needs of these fungi. Despite this, all eight strains demonstrated successful colony formation, confirming that rice malt extract powder is generally adequate for routine microbiological applications, especially when the highest possible colony densities are not required or a moderated growth rate is acceptable.

#### 3.5.2. Optimization of YM Agar Formulations Using Malt Extract Powder

This section explored the optimization of YM agar formulations utilizing rice malt extract powder as a substitute for commercial malt extract, aiming to determine the most effective formulation for supporting microbial growth. Commercial YM agar, widely employed for cultivating yeasts and molds, typically comprises malt extract, yeast extract, peptone, dextrose, and agar, delivering balanced nutrition for microbial proliferation. In this study, ten distinct YM agar formulations (F1–F10) were prepared, varying the concentrations and combinations of rice malt extract powder and other nutrients to identify formulations yielding growth results comparable to the commercial control (F10).

A range of microorganisms was used to assess each formulation, including the filamentous fungus *Aspergillus niger* TISTR5161, the yeast *Saccharomyces cerevisiae* TISTR5161, the Gram-positive bacterium *Bacillus subtilis* TISTR 001, and the Gram-negative bacterium *Escherichia coli* TISTR780. Each strain was inoculated at a concentration of 10^2^–10^3^ CFU per plate and incubated under conditions optimal for each organism. Microbial growth was evaluated both qualitatively and quantitatively, with colony counts used as the principal comparative metric.

The results, summarized in Table 13, show that certain formulations incorporating rice malt extract powder, notably F1 and F4, achieved colony counts that were statistically indistinguishable from the commercial YM agar control (F10) for all tested organisms. Specifically, F1, which directly substituted rice malt extract powder for commercial malt extract, and F4, which relied exclusively on rice malt extract powder without added dextrose, both supported robust growth of fungi, yeast, and bacteria, matching or exceeding the performance of the commercial standard. These findings suggest that rice malt extract powder provides sufficient fermentable sugars and other essential nutrients, with the inclusion of dextrose being unnecessary when using the extract, likely due to its intrinsic sugar content derived from the rice substrate [51,52]. Other formulations tested (F2–F9) supported microbial growth to varying degrees; however, in some cases (e.g., F9 for *E. coli*), colony counts were significantly lower, indicating suboptimal nutrient composition or balance.

In conclusion, the experimental analysis demonstrates that rice malt extract powder can effectively replace commercial malt extract in YM agar formulations, with certain formulations (notably F1 and F4) matching the efficacy of the commercial product across a spectrum of microbial types. These results substantiate the viability of rice malt extract powder as a functional ingredient in microbiological media, further supporting its application for routine laboratory and industrial microbial culture.

#### 3.5.3. Production of a Gluten-Free Malt Beverage with a Beer-like Flavor and No Alcohol Using Rice Malt Extract Powder

This study evaluated the feasibility of producing a gluten-free, non-alcoholic malt beverage with a beer-like profile using rice malt extract powder as the principal ingredient. The formulation process involved preparing a low-sugar wort by dissolving rice malt extract powder in water at a 1:11 ratio, resulting in a wort with an initial sugar concentration of 8°Plato. Fermentation was conducted using *Saccharomyces cerevisiae* RSU No.10 at 30 °C over 14 days. To meet the regulatory limit for non-alcoholic status (<0.5% ABV), vacuum evaporation was applied to the freshly fermented product for alcohol reduction, followed by filling and pasteurization prior to analysis [53]. The physicochemical characteristics of both the wort and the resulting beer are summarized in Table 14.

The physicochemical characteristics of both the wort and the resulting beer are summarized in Table 14. The pale malt color (EBC scale) of the wort (1.74 ± 0.05) and final beer (2.82 ± 0.15) fell within the lightest range defined for commercial pale beers, making the product visually appealing to consumers seeking a conventional beer-like appearance. Turbidity measurements decreased from 4.42 ± 0.26 EBC Helm in wort to 2.00 ± 0.22 EBC Helm in the beer, suggesting effective clarification. The specific gravity (1.0110 ± 0.0010) and residual extract (2.5 ± 0.15 °Plato) in the final product indicated the presence of unfermented or partially fermented sugars, which, together with observed reducing sugar content (see Table 15), contributed to the beverage’s distinct sweetness and mouthfeel.

The fermentation process yielded a low-alcohol malt beverage (2.58% ABV) with acceptable sensory characteristics, demonstrating the feasibility of using rice malt extract as a fermentable substrate for gluten-free malt beverage production. However, as the alcohol content exceeded the non-alcoholic threshold (<0.5% ABV), further optimization of vacuum evaporation or the application of alternative dealcoholizing methods would be necessary to develop a truly non-alcoholic product.

The wort’s pH of 5.76 ± 0.14 dropped to 4.08 ± 0.17 after fermentation, consistent with the acidification typically observed in beer fermentation and important for both taste and microbiological safety. Free amino nitrogen (FAN) decreased from 112.81 ± 11.63 mg/L in wort to 69.42 ± 10.10 mg/L in the beer, indicating nutrient consumption by yeast during fermentation, though the final value was slightly below the optimal range for yeast health, which may influence fermentation efficiency and flavor development in the final product.

To further evaluate nutritional and functional quality, the rice malt beverage was compared to a commercial non-alcoholic beer (Alcohol Free Heineken) in Table 15. The rice malt-based beer displayed higher reducing sugar content (1.32 ± 0.15 g/100 g versus 0.45 ± 0.15 g/100 g, *p* < 0.05) and slightly higher thiamin (vitamin B1) levels (0.35 ± 0.07 mg/100 g). Protein, total polyphenols, and GABA contents in the rice malt beverage were comparable or modestly lower than those in commercial control, but differences were not always statistically significant. Notably, soluble fiber content was similar in both beverages, confirming the functional integrity expected from a malt-based product.

In summary, malt extract powder from Chainat 1 rice demonstrates substantial potential as the principal substrate in gluten-free malt beverage production, offering a range of bioactive and nutritional compounds. While alcohol levels exceeded the non-alcoholic threshold in this trial, the results confirm the feasibility of formulating a gluten-free, beer-like beverage that can meet the needs of consumers with specific dietary restrictions or preferences. Future work should address process modifications required to further reduce residual alcohol and optimize sensory and nutritional properties for commercial application.

## 4. Conclusions

This study successfully demonstrated the pilot-scale production of gluten-free rice malt extract powder from the Thai Chainat 1 variety, optimized through malting and enzymatic hydrolysis. The product exhibited a favorable nutritional and functional profile, including high maltose, GABA, and B-vitamin contents, while meeting stringent safety requirements with no detectable gluten or contaminants. Shelf-life testing confirmed long-term stability across packaging formats. Application studies showed that the powder can replace conventional barley malt extract in microbiological media and serve as a functional base for gluten-free malt beverages. While the trial beverage exceeded regulatory alcohol limits, the findings highlight strong potential with further optimization of fermentation or alcohol-removal processes. Future research may focus on sensory optimization and cost-effectiveness. Overall, this work establishes technical feasibility of rice malt extract powder as a high-value, locally sourced ingredient that addresses demand for gluten-free foods, supports Thai agricultural diversification, and reduces reliance on imported malt extract.

## 5. Limitations

Limitations of this study include the inability to achieve non-alcoholic status (<0.5% ABV) in the gluten-free malt beverage formulation, and lower growth rates observed for certain filamentous fungi in rice malt extract-based media. Addressing these issues will require further process development for alcohol control and targeted media optimization to fully realize the potential of rice malt extract powder for diverse commercial applications. Moreover, shelf-life evaluation in this study was restricted to two packaging types and did not assess the potential effects of varied distribution or storage conditions, such as fluctuations in humidity and temperature during transport. The sensory evaluation of the malted rice is needed to confirm acceptance across different target markets. Furthermore, the absence of untargeted analytical platforms (e.g., LC-MS-based metabolomics) limited the comprehensive profiling of chemical composition changes during germination and processing.

## Figures and Tables

**Table 1:** Formulations of Yeast and Mold (YM) Agar Prepared with Rice Malt Extract Powder and Commercial Malt Extract (Control, F10).

Component	F1	F2	F3	F4	F5	F6	F7	F8	F9	F10 (Control)
Malt Extract Powder from Chainat 1	3.0 g	3.0 g	5.0 g	10.0 g	6.0 g	8.0 g	13.0 g	11.0 g	21.0 g	
Conventional malt extract		3.0 g	3.0 g	3.0 g						3.0 g
Yeast Extract	3.0 g		3.0 g	3.0 g		3.0 g	3.0 g			3.0 g
Peptone	5.0 g	5.0 g		5.0 g	5.0 g		5.0 g			5.0 g
Dextrose (Glucose)	10.0 g	10.0 g	10.0 g		10.0 g	10.0 g		10.0 g		10.0 g
Agar	15.0 g	15.0 g	15.0 g	15.0 g	15.0 g	15.0 g	15.0 g	15.0 g	15.0 g	15.0 g

**Table 2:** Nutritional composition of unmalted and malted Chainat 1 rice (70% polishing level).

Parameter	Unmalted (Chainat 1)	Malted (Chainat 1)
Amylose (%)	29.31 ± 1.20 ^a^	27.88 ± 0.21 ^b^
Moisture (%)	9.08 ± 0.20 ^a^	5.49 ± 0.10 ^b^
Water activity	0.476 ± 0.003 ^a^	0.284 ± 0.002 ^b^
Energy (Kcal/100 g)	354.7 ± 2.4 ^a^	350.0 ± 2.8 ^a^
GABA (mg/100 g)	2.05 ± 0.17 ^b^	10.63 ± 0.22 ^a^
Carbohydrates (g/100 g)	78.31 ± 0.85 ^a^	75.70 ± 0.55 ^b^
Protein (g/100 g)	6.95 ± 0.05 ^b^	8.88 ± 0.00 ^a^
Fat (g/100 g)	1.52 ± 0.50 ^a^	1.30 ± 0.55 ^a^
Crude fiber (g/100 g)	2.00 ± 0.56 ^b^	5.78 ± 0.55 ^a^
Total sugars (g/100 g)	2.02 ± 0.25 ^b^	11.54 ± 2.60 ^a^
Reducing sugars (g/100 g)	0.04 ± 0.00 ^b^	4.73 ± 0.32 ^a^
Glucose (g/100 g)	NT	1.37
Fructose (g/100 g)	NT	0.06
Sucrose (g/100 g)	NT	2.54
Maltose (g/100 g)	NT	3.30
Sodium (mg/100 g)	NT	3.78
Iron (mg/100 g)	NT	0.400
Calcium (mg/100 g)	NT	16.81
Vitamin B1 (mg/100 g)	0.12 ± 0.00 ^b^	0.21 ± 0.00 ^a^
Vitamin B2 (mg/100 g)	NT	0.08
Vitamin B3 (µg/100 g)	NT	10,240
Vitamin B5 (mg/100 g)	NT	1.71
Vitamin B6 (µg/100 g)	NT	520.45
Vitamin B9 (mg/100 g)	NT	0.04
Ash (g/100 g)	2.11 ± 0.16 ^b^	3.09 ± 0.25 ^a^

Note: Values are expressed as mean ± standard deviation (n = 3). Different superscript letters within the same row indicate significant differences (*p* ≤ 0.05). Nutritional values are based on a dry weight basis unless otherwise stated. NT = Not tested (analysis not performed for this parameter). The results of sugar profile and vitamin B2–B9 are based on single measurements, as reported in the Certificate of Analysis by Central Laboratory (Thailand) Co., Ltd., an ISO/IEC 17025-accredited laboratory. The results are presented based on single replicate analyses, as documented in the Certificates of Analysis under Report Nos. TRBK68/22150.

**Table 3:** Reducing sugar content (g/100 g) in Chainat 1 rice wort at β-amylase rest phase under varying time and temperature.

Sample	Reducing Sugar Content of Chainat 1 Rice Wort (g/100 g)			
	50 °C 30 min	50 °C 60 min	65 °C 30 min	65 °C 60 min
Unmalted	0.83 ± 0.06 ^ns^	0.84 ± 0.06 ^ns^	0.87 ± 0.06 ^ns^	0.84 ± 0.05 ^ns^
Malted	6.53 ± 0.05 ^b^	7.30 ± 0.02 ^a^	5.43 ± 0.39 ^c^	5.48 ± 0.40 ^c^

Note: Values are mean ± SD (n = 3). Different letters in the same row indicate statistically significant differences (*p* ≤ 0.05). Superscript ns indicates no statistically significant difference between values in this column (*p* > 0.05).

**Table 4:** Total reducing sugar content (g/100 g) in Chainat 1 rice wort under α-amylase rest at different time-temperature combinations.

Sample	Reducing Sugar Content of Chainat 1 Rice Wort (g/100 g)			
	55 °C 30 min	55 °C 60 min	75 °C 30 min	75 °C 60 min
Unmalted	0.87 ± 0.06 ^ns^	0.87 ± 0.04 ^ns^	0.86 ± 0.07 ^ns^	0.87 ± 0.05 ^ns^
Malted	7.41 ± 0.98 ^d^	8.10 ± 0.91 ^c^	12.45 ± 1.50 ^b^	14.89 ± 0.13 ^a^

Note: Values are mean ± SD (n = 3). Different letters in the same row indicate statistically significant differences (*p* ≤ 0.05). Superscript ns indicates no statistically significant difference between values in this column (*p* > 0.05).

**Table 5:** Soluble protein and fermentable sugar contents under different enzymatic extraction treatments.

Parameter	Protease/β-Amylase Rest Phase		α-Amylase Rest Phase	
	T1 (Endogenous Enzymes Only)	T2 (Added Protease & β-Amylase, 50 °C)	T3 (Added Protease & β-Amylase, no α-Amylase, 75 °C)	T4 (Full Enzyme Supplementation, with α-Amylase, 75 °C)
Soluble protein (mg/mL)	1.45 ± 0.10 ^b^	2.94 ± 0.20 ^a^	2.90 ± 0.23 ^a^	2.87 ± 0.23 ^a^
Maltose (g/100 g)	5.16 ± 0.21 ^c^	12.48 ± 0.26 ^b^	12.45 ± 0.20 ^b^	14.62 ± 0.15 ^a^
Glucose (g/100 g)	1.75 ± 0.05 ^c^	1.70 ± 0.06 ^c^	3.82 ± 0.10 ^b^	5.01 ± 0.10 ^a^
Sucrose (g/100 g)	ND	ND	ND	0.48 ± 0.02
Fructose (g/100 g)	ND	ND	ND	0.02 ± 0.00
Xylose (g/100 g)	0.39 ± 0.09 ^b^	0.40 ± 0.05 ^b^	0.45 ± 0.02 ^b^	1.46 ± 0.02 ^a^

Note: Values are mean ± SD (n = 3). Different letters in the same row indicate statistically significant differences (*p* ≤ 0.05). ND = Not detected (below Limit of Detection).

**Table 6:** Nutritional composition of rice malt extract powder produced from malted Chainat 1 rice (per 100 g, dry weight basis).

Items	Unit	Results (1)	Reference Method	Results * (2)	LOD
Energy (kcal)	kcal/100 g	284.2 ± 25.0	Calculation	395.70 **	
Carbohydrate	g/100 g	88.9 ± 6.4	Calculation	92.60 **	
Protein	g/100 g	5.20 ± 0.55	AOAC (2000) 981.10 [23]	5.20	-
Total fat	g/100 g	0.48 ± 0.50	AOAC (2000) 922.06 [23]	0.50	-
Saturated fat	g/100 g	NT	AOAC (2023) 996.06 [37]	0.22	
Cholesterol	mg/100 g	NT		0.00	0.50
Ash	g/100 g	0.367 ± 0.150	AOAC (2000) 920.153 [23]	0.39	-
Moisture	g/100 g	1.74 ± 0.05	AOAC (2000) 925.45(A) [23]	1.65	-
Total Sugar	g/100 g	64.77 ± 4.55		59.62	0.03
Maltose	g/100 g	NT		43.85	0.30
Glucose	g/100 g	NT		14.31	0.30
Sucrose	g/100 g	NT		1.46	0.30
Fructose	g/100 g	NT		<0.30	0.30
Lactose	g/100 g	NT		ND	0.30
Xylose	g/100 g	4.38 ± 0.50		NT	
GABA	mg/100 g	245.20 ± 2.55	[8,11]	NT	
Total Phenolic	mgGAE/100 g	25.59 ± 0.26	[21,22]	NT	
Phytic acid	mg/100 g	2105.60 ± 11.4	[8]	NT	
Vitamin B1	mg/100 g	0.64 ± 0.07	[16]	NT	
Potassium	mg/100 g	73.85 ± 4.15	[12]	78.24 ***	
Sodium	mg/100 g	39.46 ± 5.02	[12]	43.55	-
Magnesium	mg/100 g	91.96 ± 5.55	[12]	NT	
Calcium	mg/100 g	161.76 ± 15.2	[12]	NT	

Result (1): Results quantified in faculty of food technology, Rangsit University, Thailand. Result (2): Results certified by Central Laboratory (Thailand) Co., Ltd., an ISO/IEC 17025-accredited laboratory [28]. * Reported the results in the Certificate of Analysis by Central Laboratory (Thailand) Co., Ltd., an ISO/IEC 17025-accredited laboratory. ** Reference method: In-house method TE-CH-169 based on Method of Analysis for Nutrition Labeling [38]. *** Reference method: AOAC (2023) 996.06 [37], ND = Not Detected (below Limit of Detection), NT = Not Tested (analysis not performed for this parameter).

**Table 7:** Amino acid profile (mg/100 g) of rice malt extract powder from malted Chainat 1 rice.

Amino Acid	Amount (mg/100 g)	LOD (mg/100 g)	Reference Method
Aspartic acid	474.91	100.00	CH-372 based on Official Journal of The European Communities Directive 98/64/EC L257(1998), Annex Part A [41]
Threonine	<200.00	100.00	
Serine	259.43	100.00	
Glutamic acid	863.49	50.00	
Glycine	214.51	50.00	
Alanine	264.68	50.00	
Cystine	<200.00	100.00	
Valine	285.24	50.00	
Methionine	<100.00	100.00	
Isoleucine	166.59	50.00	
Leucine	358.62	50.00	
Tyrosine	<250.00	100.00	
Phenylalanine	<250.00	100.00	
Histidine	<100.00	100.00	
Hydroxylysine	ND	100.00	
Lysine	185.79	50.00	
Tryptophan	ND		
Arginine	388.36	100.00	
Proline	214.30	100.00	

Note: The results of amino acid assessment are based on single measurements, as reported in the Certificate of Analysis by Central Laboratory (Thailand) Co., Ltd., an ISO/IEC 17025-accredited laboratory. The results are presented based on single replicate analyses, as documented in the Certificates of Analysis under Report Nos. TRBK68/00028. ND = Not Detected (below Limit of Detection). LOD = Limit of Detection and value are indicated where applicable.

**Table 8:** Microbiological quality assessment of malt extract powder from Chainat 1 rice.

Item	Result	Unit	Reference Method
*Staphylococcus aureus*	<10 est.	CFU/g	AOAC 2023 2003.07 [37]
Yeast and Mold	<10 est.	CFU/g	AOAC 2023 997.02 [37]
*Bacillus cereus*	<10	CFU/g	ISO 7932:2004 [43] and FDA BAM Online, 2020 (Chapter 14) [44]
*Clostridium perfringens*	<10	CFU/g	ISO 15213-2:2023 [45]
Coliforms	2.2	MPN/100 mL	In-house method TE–MI-171 based on Standard Methods for the Examination of Water and Wastewater, APHA, AWWA, WEF, 24th Ed., 2023. Part 9221B [46]
*Escherichia coli*	ND	per 100 mL	In-house method TE–MI-171 based on Standard Methods, Part 9221B, and 9221F [46]
*Salmonella* spp.	ND	per 25 g	ISO 6579-1:2017/Amd1:2020 [47]

Note: The results of microbiological assessment are based on single measurements, as reported in the Certificate of Analysis by Central Laboratory (Thailand) Co., Ltd., an ISO/IEC 17025-accredited laboratory. The results are presented based on single replicate analyses, as documented in the Certificates of Analysis under Report Nos. TRBK68/00027 Part 2. ND = Not detected (below Limit of Detection), LOD = Limit of Detection.

**Table 9:** Contaminant analysis of rice malt extract powder: mycotoxins, gluten, and heavy metals.

Item	Result	Unit	LOD	Reference Method
Aflatoxin B1	ND	µg/kg	0.25	In-house method TE-CHI-025 based on AOAC (2023) 991.31 and 994.08 [37]
Aflatoxin B2	ND	µg/kg	0.25	
Aflatoxin G1	ND	µg/kg	0.25	
Aflatoxin G2	ND	µg/kg	0.25	
Total Aflatoxin	ND	µg/kg		
Gluten Allergen (Gliadin)	ND	mg/kg	0.85	MloBS Wheat/Gluten (Gliadin) ELISA Kit II Cat.#M2114
Arsenic	ND	mg/kg	0.025	In-house method TE-CH-134 based on AOAC (2023) 986.15 [37] by ICP-MS
Cadmium	<0.010	mg/kg	0.005	In-house method TE-CH-134 based on AOAC (2023) 999.10 [37] by ICP-MS
Lead	<0.015	mg/kg	0.010	In-house method TE-CH-134 based on AOAC (2023) 999.10 [37] by ICP-MS
Mercury	ND	mg/kg	0.005	In-house method TE-CH-134 based on AOAC (2023) 974.10 [37] by ICP-MS
Tin	ND	mg/kg	3.000	In-house method TE-CH-134 based on AOAC (2023) 985.16 [37] by ICP-MS

Note: The results of microbiological assessment are based on single measurements, as reported in the Certificate of Analysis by Central Laboratory (Thailand) Co., Ltd., an ISO/IEC 17025-accredited laboratory. The results are presented based on single replicate analyses, as documented in the Certificates of Analysis under Report Nos. TRBK68/00027 Part 1. ND = Not detected (below Limit of Detection), LOD = Limit of Detection.

**Table 10:** Stability and changes in color (L) of malt extract powder stored in laminated bags at 30 °C, 45 °C, and 55 °C.

	Color (L*)		
Day	30 °C	45 °C	55 °C
0	89.8 ± 1.05 ^aA^	89.8 ± 1.05 ^aA^	89.8 ± 1.05 ^aA^
27	90.1 ± 0.85 ^aA^	89.9 ± 1.08 ^aA^	88.5 ± 1.04 ^aA^
54	88.7 ± 1.20 ^aA^	89.6 ± 1.01 ^aA^	**77.2 ± 1.00 ^bB^**
81	88.8 ± 1.14 ^aA^	88.9 ± 1.25 ^aA^	**77.5 ± 1.35 ^bB^**
108	89.2 ± 0.95 ^aA^	87.8 ± 1.50 ^aA^	**76.2 ± 1.00 ^bB^**
135	88.8 ± 1.06 ^aA^	**77.5 ± 1.21 ^bB^**	**77.6 ± 1.30 ^bB^**
153	88.5 ± 1.00 ^aA^	**77.5 ± 1.25 ^bB^**	**77.5 ± 1.00 ^bB^**

Note: Values are expressed as mean ± standard deviation (n = 3). Different lowercase letters in the same column indicate statistically significant differences over storage time (*p* ≤ 0.05). Different uppercase letters in the same row indicate statistically significant differences between storage temperatures (*p* ≤ 0.05). Only stability parameters with significant variation (*p* ≤ 0.05) overtime or temperature were retained in the main text. Complete time-series data for all packaging conditions and temperatures are provided in Table S1 (see Supplementary Materials, Section S2).

**Table 11:** Stability and changes in vitamin B1 in rice malt extract powder stored in HDPE containers at 30 °C, 45 °C, and 55 °C.

	Vitamin B1		
Day	30 °C	45 °C	55 °C
0	0.64 ± 0.07 ^aA^	0.64 ± 0.07 ^aA^	0.64 ± 0.07 ^aA^
27	0.64 ± 0.02 ^aA^	0.63 ± 0.08 ^aA^	0.50 ± 0.04 ^aA^
54	0.68 ± 0.02 ^aA^	0.63 ± 0.04 ^aA^	**0.44 ± 0.05 ^bB^**
81	0.68 ± 0.02 ^aA^	0.62 ± 0.02 ^aA^	**0.42 ± 0.05 ^bB^**
135	0.67 ± 0.05 ^aA^	0.62 ± 0.05 ^aA^	**0.45 ± 0.03 ^bB^**
171	0.65 ± 0.05 ^aA^	0.61 ± 0.04 ^aA^	**0.43 ± 0.04 ^bB^**
180	0.63 ± 0.02 ^aA^	0.60 ± 0.06 ^aA^	**0.40 ± 0.04 ^bB^**

Note: Values are expressed as mean ± standard deviation (n = 3). Different lowercase letters in the same column indicate statistically significant differences over storage time (*p* ≤ 0.05). Different uppercase letters in the same row indicate statistically significant differences between storage temperatures (*p* ≤ 0.05). Only stability parameters with significant variation (*p* ≤ 0.05) overtime or temperature were retained in the main text. Complete time-series data for all packaging conditions and temperatures are provided in Table S2 (see Supplementary Materials, Section S3).

**Table 12:** Fungal colony counts on malt agar media formulated with rice malt extract powder versus commercial malt extract powder.

Fungal Strain	Colony Count (CFU/mL) on Medium	
	Prepared Rice Malt Extract	with Commercial Malt Extract
*Candida albicans* TISTR5554	(2.63 ± 0.15) × 10^2 ns^	(2.87 ± 0.12) × 10^2 ns^
*Saccharomyces cerevisiae* TISTR5774	(2.78 ± 0.20) × 10^2 ns^	(2.85 ± 0.10) × 10^2 ns^
*Saccharomyces cerevisiae* TISTR5240	(2.65 ± 0.32) × 10^2 ns^	(2.87 ± 0.15) × 10^2 ns^
*Geotrichum candidum* TISTR3422	(1.00 ± 0.50) × 10^2 ns^	(1.30 ± 0.05) × 10^2 ns^
*Rhodotorula mucilaginosa* TISTR5864	(2.94 ± 0.28) × 10^2 ns^	(3.02 ± 0.10) × 10^2 ns^
*Pennicillium roquefortii* TISTR3511	(1.50 ± 0.20) × 10^3 b^	(3.00 ± 0.50) × 10^3 a^
*Aspergillus niger* TISTR1867	(1.97 ± 0.85) × 10^3 b^	(3.93 ± 0.51) × 10^3 a^
*Trichoderma harzianum* TISTR3553	(1.20 ± 0.50) × 10^3 b^	(2.32 ± 0.80) × 10^3 a^

Note: Values represent mean ± standard deviation (n = 3). Different superscript letters in the same row indicate significant differences (*p* < 0.05). Superscript ns indicates no statistically significant difference between values in this column (*p* > 0.05).

**Table 13:** Growth comparison of selected microorganisms on yeast and mold agar formulated with rice malt extract powder.

Formula	Microorganism			
	*Aspergillus niger* TISTR5161	*Saccharomyces cerevisiae* TISTR5161	*Bacillus subtilis* TISTR 001	*Escherichia coli* TISTR780
F1	(1.72 ± 0.38) × 10^2 ns^	(2.55 ± 0.18) × 10^2 ns^	(1.80 ± 0.27) × 10^2 ns^	(1.80 ± 0.10) × 10^2 a^
F2	(1.62 ± 0.41) × 10^2 ns^	(2.30 ± 0.25) × 10^2 ns^	(1.71 ± 0.31) × 10^2 ns^	(1.68 ± 0.25) × 10^2 ab^
F3	(1.51 ± 0.44) × 10^2 ns^	(2.22 ± 0.31) × 10^2 ns^	(1.65 ± 0.30) × 10^2 ns^	(1.63 ± 0.22) × 10^2 ab^
F4	(1.83 ± 0.50) × 10^2 ns^	(2.66 ± 0.25) × 10^2 ns^	(1.82 ± 0.25) × 10^2 ns^	(1.98 ± 0.11) × 10^2 a^
F5	(1.45 ± 0.42) × 10^2 ns^	(2.53 ± 0.20) × 10^2 ns^	(1.62 ± 0.30) × 10^2 ns^	(1.77 ± 0.20) × 10^2 ab^
F6	(1.71 ± 0.36) × 10^2 ns^	(2.32 ± 0.16) × 10^2 ns^	(1.53 ± 0.26) × 10^2 ns^	(1.64 ± 0.24) × 10^2 ab^
F7	(1.80 ± 0.35) × 10^2 ns^	(2.50 ± 0.13) × 10^2 ns^	(1.87 ± 0.34) × 10^2 ns^	(1.67 ± 0.15) × 10^2 ab^
F8	(1.66 ± 0.41) × 10^2 ns^	(2.47 ± 0.18) × 10^2 ns^	(1.77 ± 0.27) × 10^2 ns^	(1.52 ± 0.35) × 10^2 ab^
F9	(1.80 ± 0.55) × 10^2 ns^	(2.28 ± 0.28) × 10^2 ns^	(1.74 ± 0.35) × 10^2 ns^	(1.06 ± 0.15) × 10^2 b^
F10 (Control)	(1.75 ± 0.45) × 10^2 ns^	(2.62 ± 0.21) × 10^2 ns^	(1.85 ± 0.32) × 10^2 ns^	(1.75 ± 0.15) × 10^2 a^

Note: Values represent mean ± standard deviation (n = 3). Different superscript letters in the same column indicate significant differences (*p* < 0.05). Superscript ns indicates no statistically significant difference between values in this column (*p* > 0.05). F10 = Commercial YMA medium.

**Table 14:** Physical and chemical properties of wort and beer produced using rice malt extract powder.

Analyze	Wort		Beer	
	Wort from the Rice Malt Extract Powder	EBC Criteria of Wort [54]	Beer from the Rice Malt Extract Powder	EBC Criteria of Beer [55,56]
Pale Malt Color (°EBC)	1.74 ± 0.05	4–12	2.82 ± 0.15	≤8 (pale), 8–25 (amber), >25 (dark)
Turbidity (EBC Helm)	4.42 ± 0.26	≤5.0 NTU (bright wort)	2.00 ± 0.22	≤1.5 NTU (bright beer)
Mass (°Plato)	8.0 ± 0.11	10–15°P	2.5 ± 0.15	2–6°P (light), >12°P (strong)
pH	5.76 ± 0.14	5.2–5.6	4.08 ± 0.17	4.2–4.6
FAN (mg/L)	112.81 ± 11.63	150–250 mg/L	69.42 ± 10.10	120–220 mg/L
% Extract	59.62 ± 2.50	75–82%	-	Varies by beer style
Specific gravity	1.0318 ± 0.0012	1.040–1.060	1.011 ± 0.001	1.008–1.016
% Alcohol by Vol.	0.0 ± 0.0	0.0%	2.58 ± 0.10	3.5–7.5% (typical beers)
Bitterness (BU)	0.00 ± 0.00	No standard	18.50 ± 0.55	15–35 BU

Note: Values represent mean ± standard deviation (n = 3).

**Table 15:** Nutritional, physical, and bioactive property comparison between project-derived and commercial non-alcoholic beers.

Analyze	Commercial Beer *	Produced Beer
Protein (g/100 g)	0.96 ± 0.07 ^a^	0.47 ± 0.05 ^b^
Reducing sugar (g/100 g)	0.45 ± 0.15 ^b^	1.32 ± 0.15 ^a^
Thiamin (mg/100 g)	0.32 ± 0.02 ^ns^	0.35 ± 0.07 ^ns^
Total Polyphenol (mgGAE/100 g)	0.71 ± 0.05 ^a^	0.50 ± 0.06 ^b^
GABA (mg/100 g)	4.37 ± 0.05 ^a^	4.07 ± 0.05 ^b^
Soluble Fiber (g/100 g)	0.62 ± 0.05 ^ns^	0.67 ± 0.05 ^ns^

Note: Values represent mean ± standard deviation (n = 3). Different superscript letters in the same row indicate significant differences (*p* < 0.05). Superscript ns indicates no statistically significant difference between values in this column (*p* > 0.05). * Commercial non-alcoholic beer used for comparison was Alcohol-Free Heineken (Thai Asia Pacific Brewery Co., Ltd., Nonthaburi, Thailand) (production lot: L4342702E, time stamp: 11:59).

## Data Availability

The data presented in this study are available on request from the corresponding author (the data are not publicly available due to privacy or ethical restrictions).

## Supplementary Materials

The following supporting information can be downloaded at: https://www.mdpi.com/article/10.3390/molecules30214279/s1, Section S1: HPLC chromatogram for extracted solution; Section S2: Shelf life for malt extract powder from Chainat 1 rice packed in laminated pouch; Section S3: Shelf life for malt extract powder from Chainat 1 rice packed in HDPE container; Figure S1 shows the chromatogram of extract obtained at 50 °C for 60 min without supplementation of protease and β-amylase. Figures S2 and S3 display chromatograms of extracts obtained at 75 °C for 60 min, without and with the supplementation of α-amylase, respectively. Table S1 summarizes the stability of color (L* value), moisture, and total phenolic content of rice malt extract powder stored in laminated bags at 30 °C, 45 °C, and 55 °C. Table S2 presents the stability of color (L* value), moisture, total phenolics, and vitamin B_1_ of rice malt extract powder stored in HDPE containers under the same temperature conditions.

## Abbreviations

The following abbreviations are used in this manuscript:

ANOVA	Analysis of Variance
AOAC	Association of Official Analytical Chemists
°Brix	Degrees Brix (sugar content of an aqueous solution)
CFU	Colony-Forming Unit
CFU/mL	Colony-Forming Units per milliliter (specific to microbial counts)
DM	Dry Matter (sometimes used instead of DW)
DP	Diastatic Power
DW	Dry Weight
FAO	Food and Agriculture Organization
FDA	Food and Drug Administration
GABA	Gamma-Aminobutyric Acid
GC–MS	Gas Chromatography–Mass Spectrometry
GE	Germination Energy
HDPE	High-Density Polyethylene
HPLC	High-Performance Liquid Chromatography
H_2_SO_4_	Sulfuric acid
ICP–MS	Inductively Coupled Plasma–Mass Spectrometry
ISO	International Organization for Standardization
LOD	Limit of Detection
LOQ	Limit of Quantification
1-MCP	1-Methylcyclopropene (ripening inhibitor, mentioned in storage trials)
MRS	de Man, Rogosa, and Sharpe medium
NA	Not Analyzed/Not Applicable
NaOH	Sodium hydroxide
ND	Not Detected
NIST	National Institute of Standards and Technology
PBS	Phosphate-Buffered Saline
PCA	Plate Count Agar
°Plato (°P)	Degrees Plato (percentage of extract in wort by weight)
RH	Relative Humidity
SD	Standard Deviation
SEM	Standard Error of the Mean (sometimes appears alongside SD)
SPSS	Statistical Package for the Social Sciences
TPC	Total Plate Count
UV–Vis	Ultraviolet–Visible Spectrophotometry
WHO	World Health Organization
