# Supplementary material for: Gluten-Free Rice Malt Extract Powder: Pilot-Scale Production, Characterization, and Food Applications
Source: Molecules. 2025 Nov 3;30(21):4279. doi: 10.3390/molecules30214279 (PMC12610662; doi:10.3390/molecules30214279)
Supplement: Supplementary file 1 [file molecules-30-04279-s001.zip › molecules-3859261-supplementary.pdf]

SUPPLEMENTARY MATERIALS

Gluten-Free Rice Malt Extract Powder: Pilot-Scale Production, Characterization, and Food Applications

Corresponding Author: Yupakanit Phuangweerakul

SECTION S1: HPLC chromatogram for extracted solution

Figure S1-S3 presents chromatographic profiles obtained from High-Performance Liquid Chromatography (HPLC) analysis of malt extract solutions under different extraction conditions. Each represents a distinct combination of extraction temperature, duration, and enzyme supplementation:

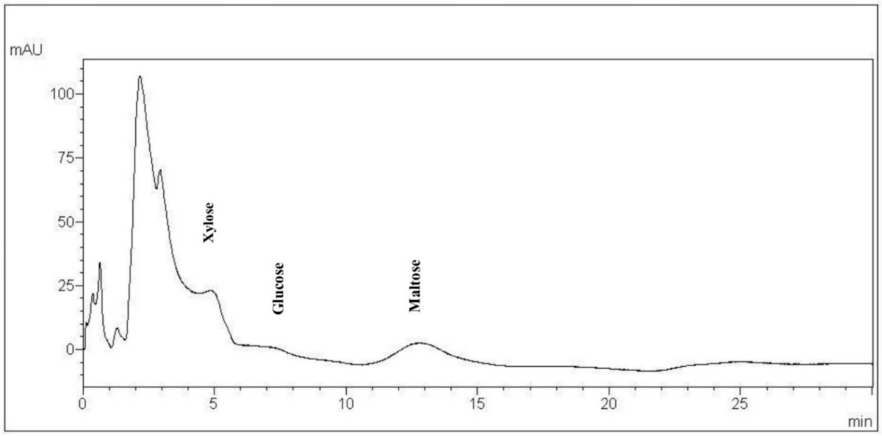

Figure S1. HPLC chromatogram of malt extract solution obtained at 50 °C for 60 minutes without supplementation of protease and β-amylase.

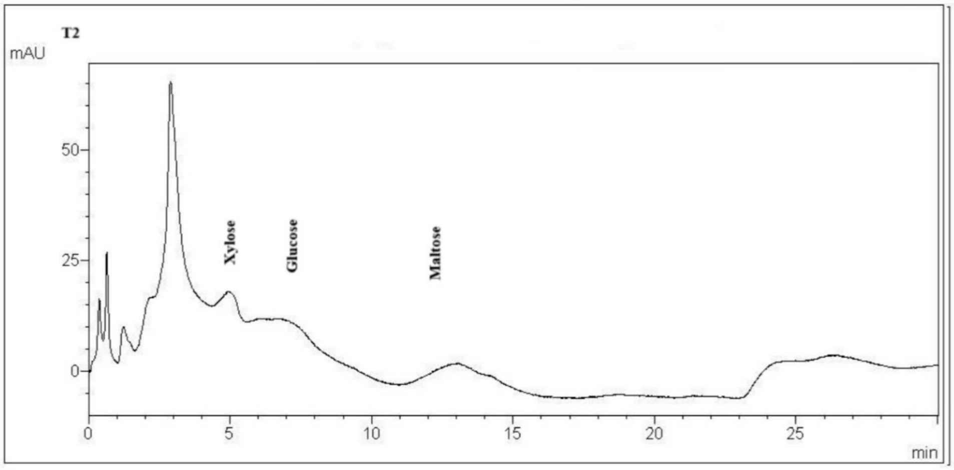

Figure S2. HPLC chromatogram of malt extract solution obtained at 75 °C for 60 minutes without supplementation of α-amylase.

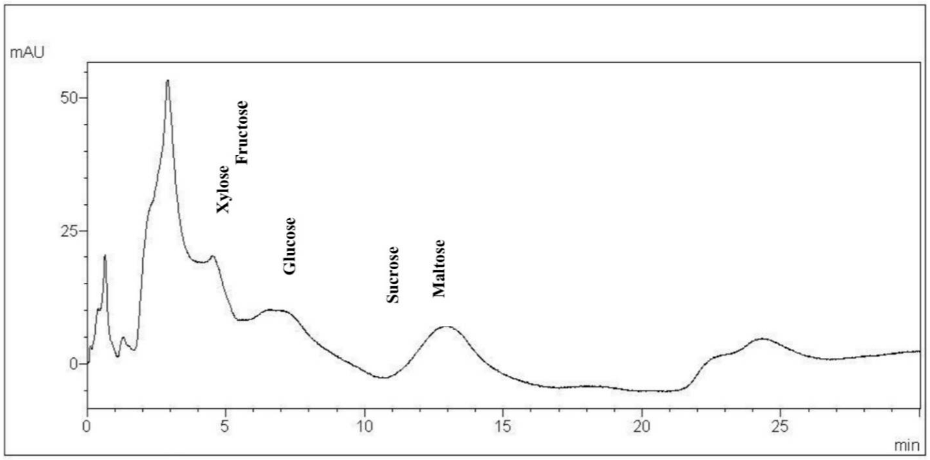

Figure S3. HPLC chromatogram of malt extract solution obtained at 75 °C for 60 minutes with supplementation of α-amylase.

These chromatograms illustrate differences in peak intensity and retention times, indicating variations in the concentration and composition of extracted compounds depending on the extraction temperature and the presence or absence of specific enzymes. Comparative presentation allows assessment of how enzymatic treatment influences the breakdown of malt components and the profile of bioactive or nutritional compounds in the extract.

SECTION S2: Shelf life for malt extract powder from Chainat 1 rice packed in laminated pouch

Section S2.1 *Changes in properties of malt extract powder from Chainat 1 rice packed in packed in laminated pouch over the study period.*

Table S1. Stability of color (L), moisture, and total phenolic content of rice malt extract powder stored in **laminated bags** at 30°C, 45°C, and 55°C.

	Color (L*)			Moisture content		
Day	30°C	45°C	55°C	30°C	45°C	55°C
0	89.8±1.05^{aA}	89.8±1.05^{aA}	89.8±1.05^{aA}	1.74±0.05^{aA}	1.74±0.05^{aA}	1.74±0.05^{aA}
27	90.1±0.85^{aA}	89.9±1.08^{aA}	88.5±1.04^{aA}	1.74±0.01^{aA}	1.74±0.01^{aA}	1.76±0.02^{abA}
54	88.7±1.20^{aA}	89.6±1.01^{aA}	**77.2±1.00^{bB}**	1.77±0.03^{aB}	1.76±0.04^{aB}	1.79±0.01^{bA}
81	88.8±1.14^{aA}	88.9±1.25^{aA}	**77.5±1.35^{bB}**	1.76±0.02^{aB}	1.77±0.05^{aB}	1.79±0.00^{aA}
108	89.2±0.95^{aA}	87.8±1.50^{aA}	**76.2±1.00^{bB}**	1.76±0.04^{aA}	1.78±0.01^{aA}	1.80±0.02^{aA}
135	88.8±1.06^{aA}	**77.5±1.21^{bB}**	**77.6±1.30^{bB}**	1.77±0.01^{aB}	1.80±0.00^{bA}	1.83±0.03^{aA}
153	88.5±1.00^{aA}	**77.5±1.25^{bB}**	**77.5±1.00^{bB}**	1.75±0.05^{aC}	1.79±0.00^{abB}	1.84±0.02^{aA}
	**DPPH**			**Total Phenolic Content (mgGAE/100g)**		
Day	30°C	45°C	55°C	30°C	45°C	55°C
0	22.90±0.56aA	22.90±0.56aA	22.90±0.56aA	5.59±0.26^{aA}	5.59±0.26^{aA}	5.59±0.26^{aA}
27	22.91±0.40aA	23.02±0.48aA	22.91±0.58aA	5.57±0.27^{aA}	5.59±0.30^{aA}	5.54±0.32^{aA}
54	22.51±0.66aA	22.88±0.41aA	22.86±0.74aA	5.57±0.42^{aA}	5.53±0.30^{aA}	5.50±0.46^{aA}
81	22.40±0.79aA	22.67±0.32aA	22.36±0.49aA	5.55±0.45^{aA}	5.38±0.48^{aA}	5.51±0.28^{aA}
108	22.36±1.10aA	22.48±0.65aA	22.20±0.62aA	5.57±0.48^{aA}	5.48±0.13^{aA}	5.50±0.37^{aA}
135	22.33±0.72aA	22.48±0.44aA	22.35±0.66aA	5.59±0.55^{aA}	5.33±0.57^{aA}	5.39±0.34^{aA}
153	22.41±0.47aA	22.33±0.50aA	22.30±0.50aA	5.57±0.57^{aA}	5.39±0.26^{aA}	5.27±0.53^{aA}

Note: Values are expressed as mean ± standard deviation (n = 3).
Different lowercase letters in the same column indicate statistically significant differences over storage time ($p \leq 0.05$).
Different uppercase letters in the same row indicate statistically significant differences between storage temperatures ($p \leq 0.05$).

Section S2.2: Calculation of shelf life for malted rice extract powder storage in laminated pouchs

The shelf-life estimation of the malted rice extract powder using the **Q_{10} method** under accelerated storage conditions. The Q_{10} value represents the factor by which the reaction rate changes when the temperature increases by 10 °C. Shelf-life predictions were based on the degradation rate of vitamin B_1, which served as the quality indicator.

Laminate pouchs	
$Q10 = \theta S(T)/\ \theta S(T+10)$<br>$= \theta S(45)/\ \theta S(55)$<br>$= 135/54$<br>$= 2.5$<br>$Q1 = \theta 10^{0.1}$<br>$= 2.5^{0.1}$<br>$= 1.096$<br>$Q145\text{-}30 = 1.096^{15}$<br>$= \theta S(30)/\ \theta S(45)$<br>$= \theta S(30)/135$<br>$\theta S(30) = 1.096^{15} x135$<br>$= 534$ days	For the **laminate packaging**, the degradation rate constant (θ_s) was determined at 45 °C and 55 °C, yielding a Q_{10} value of 2.5. From this, the rate constant adjustment factor (Q_1) for a 1 °C temperature difference was calculated as 1.096. Applying the Q_1 factor for the temperature difference between 30 °C and 45 °C resulted in a predicted shelf life of approximately **534 days** at 30 °C.

SECTION S3: Shelf life for malt extract powder from Chainat 1 rice packed in HDPE container

***Section S3.1** Changes in properties of malt extract poweder from Chainat 1 rice packed in HDPE container over the study period.*

Table S2 Stability of color (L), moisture, total phenolics, and vitamin B1 in rice malt extract powder stored in **HDPE containers** at 30°C, 45°C, and 55°C.

	Color (L*)			Moisture content		
**Day**	**30°C**	**45°C**	**55°C**	**30°C**	**45°C**	**55°C**
0	89.8 ± 1.05^{aA}	89.8 ± 1.05^{aA}	89.8 ± 1.05^{aA}	1.76 ± 0.10^{aA}	1.76 ± 0.14^{aA}	1.76 ± 0.15^{aA}
27	90.1 ± 0.85^{aA}	89.9 ± 1.08^{aA}	88.5 ± 1.04^{aA}	1.77 ± 0.09^{aA}	1.75 ± 0.11^{aA}	1.76 ± 0.12^{aA}
54	88.7 ± 1.20^{aA}	89.6 ± 1.01^{aA}	88.9 ± 1.06^{aA}	1.74 ± 0.11^{aA}	1.77 ± 0.10^{aA}	1.77 ± 0.10^{aA}
81	88.8 ± 1.14^{aA}	88.9 ± 1.25^{aA}	88.8 ± 1.10^{aA}	1.77 ± 0.15^{aA}	1.76 ± 0.15^{aA}	1.74 ± 0.10^{aA}
135	89.2 ± 0.95^{aA}	87.8 ± 1.50^{aA}	89.9 ± 1.06^{aA}	1.76 ± 0.14^{aA}	1.77 ± 0.12^{aA}	1.78 ± 0.15^{aA}
171	88.8 ± 1.06^{aA}	89.8 ± 1.02^{aA}	88.8 ± 1.00^{aA}	1.77 ± 0.11^{aA}	1.72 ± 0.15^{aA}	1.78 ± 0.10^{aA}
180	89.5 ± 1.05^{aA}	89.2 ± 1.00^{aA}	89.4 ± 1.05^{aA}	1.75 ± 0.15^{aA}	1.75 ± 0.15^{aA}	1.77 ± 0.15^{aA}
	**Vitamin B1**			**Total Phenolic Content (mgGAE/100g)**		
**Day**	**30°C**	**45°C**	**55°C**	**30°C**	**45°C**	**55°C**
0	0.64 ± 0.07^{aA}	0.64 ± 0.07^{aA}	0.64 ± 0.07^{aA}	5.36 ± 0.58^{aA}	5.36 ± 0.56^{aA}	5.36 ± 0.56^{aA}
27	0.64 ± 0.02^{aA}	0.63 ± 0.08^{aA}	0.50 ± 0.04^{aA}	5.43 ± 0.27^{aA}	5.40 ± 0.52^{aA}	5.45 ± 0.50^{aA}
54	0.68 ± 0.02^{aA}	0.63 ± 0.04^{aA}	$\mathbf{0.44\pm0.05^{bB}}$	5.38 ± 0.29^{aA}	5.48 ± 0.28^{aA}	5.38 ± 0.35^{aA}
81	0.68 ± 0.02^{aA}	0.62 ± 0.02^{aA}	$\mathbf{0.42\pm0.05^{bB}}$	5.49 ± 0.40^{aA}	5.49 ± 0.51^{aA}	5.51 ± 0.32^{aA}
135	0.67 ± 0.05^{aA}	0.62 ± 0.05^{aA}	$\mathbf{0.45\pm0.03^{bB}}$	5.52 ± 0.58^{aA}	5.50 ± 0.43^{aA}	5.50 ± 0.46^{aA}
171	0.65 ± 0.05^{aA}	0.61 ± 0.04^{aA}	$\mathbf{0.43\pm0.04^{bB}}$	5.53 ± 0.44^{aA}	5.52 ± 0.57^{aA}	5.51 ± 0.55^{aA}
180	0.63 ± 0.02^{aA}	0.60 ± 0.06^{aA}	$\mathbf{0.40\pm0.04^{bB}}$	5.47 ± 0.32^{aA}	5.45 ± 0.54^{aA}	5.47 ± 0.56^{aA}

Note: Values are expressed as mean ± standard deviation (n = 3).
Different lowercase letters in the same column indicate statistically significant differences over storage time ($p \leq 0.05$).
Different uppercase letters in the same row indicate statistically significant differences between storage temperatures ($p \leq 0.05$)

Section S3.2: Calculation of shelf life for malted rice extract powder storage in HDPE container

The shelf-life estimation of the malted rice extract powder using the **Q_{10} method** under accelerated storage conditions. The Q_{10} value represents the factor by which the reaction rate changes when the temperature increases by 10 °C. Shelf-life predictions were based on the degradation rate of vitamin B_1, which served as the quality indicator.

The shelf life for the malted rice extract powder, determined based on the decrease in vitamin B_1 content under accelerated storage conditions, was used to predict the storage period.

HDPE		
Q10	=	θS(T)/ θS(T+10)
	=	θS(45)/ θS(55)
	=	180/54
	=	3.3
Q1	=	$\theta 10^{0.1}$
	=	$3.3^{0.1}$
	=	1.128
$Q1^{45\text{-}30}$	=	1.128^{15}
	=	θS(30)/ θS(45)
	=	θS(30)/180
θS(30)	=	1.128^{15}x180
	=	1,095 days

For the **HDPE packaging**, θ_s values at 45 °C and 55 °C gave a Q_{10} value of 3.3, corresponding to a Q_1 factor of 1.128. When adjusted for the temperature difference between 30 °C and 45 °C, the predicted shelf life at 30 °C was **1,095 days** (approximately three years).

These results indicate that **HDPE packaging provides superior protection** for the malted rice extract powder compared to laminate packaging, effectively doubling the predicted shelf life under ambient conditions. However, the predictions are based on controlled accelerated storage and may not fully reflect variations in real distribution and storage environments.
